# An alternative pathway to plant cold tolerance in the absence of vacuolar invertase activity

**DOI:** 10.1111/tpj.16049

**Published:** 2022-12-22

**Authors:** Paula Teper‐Bamnolker, Marina Roitman, Omri Katar, Noam Peleg, Kalaivani Aruchamy, Shlomit Suher, Adi Doron‐Faigenboim, Diana Leibman, Ayelet Omid, Eduard Belausov, Mariette Andersson, Niklas Olsson, Ann‐Sofie Fält, Hanne Volpin, Per Hofvander, Amit Gal‐On, Dani Eshel

**Affiliations:** ^1^ Department of Postharvest Science, Agricultural Research Organization (ARO) The Volcani Institute Rishon LeZion Israel; ^2^ The Robert H. Smith Faculty of Agriculture, Food and Environment, Institute of Plant Sciences and Genetics in Agriculture The Hebrew University of Jerusalem Rehovot 76100 Israel; ^3^ Institute of Plant Sciences, Agricultural Research Organization (ARO) The Volcani Institute Rishon LeZion Israel; ^4^ Department of Plant Pathology and Weed Research, Agricultural Research Organization (ARO) The Volcani Institute Rishon LeZion Israel; ^5^ Danziger Innovations Limited Mishmar Hashiva Israel; ^6^ Department of Ornamental Horticulture, Agricultural Research Organization (ARO) The Volcani Institute Rishon LeZion Israel; ^7^ Department of Plant Breeding Swedish University of Agricultural Sciences Alnarp Sweden

**Keywords:** cold‐induced sweetening, cold stress, CRISPR/Cas9, potato, *Solanum tuberosum*, transcriptome, vacuolar invertase

## Abstract

To cope with cold stress, plants have developed antioxidation strategies combined with osmoprotection by sugars. In potato (*Solanum tuberosum*) tubers, which are swollen stems, exposure to cold stress induces starch degradation and sucrose synthesis. Vacuolar acid invertase (VInv) activity is a significant part of the cold‐induced sweetening (CIS) response, by rapidly cleaving sucrose into hexoses and increasing osmoprotection. To discover alternative plant tissue pathways for coping with cold stress, we produced *VInv‐*knockout lines in two cultivars. Genome editing of *VInv* in ‘Désirée’ and ‘Brooke’ was done using stable and transient expression of CRISPR/Cas9 components, respectively. After storage at 4°C, sugar analysis indicated that the knockout lines showed low levels of CIS and maintained low acid invertase activity in storage. Surprisingly, the tuber parenchyma of *vinv* lines exhibited significantly reduced lipid peroxidation and reduced H_2_O_2_ levels. Furthermore, whole plants of *vinv* lines exposed to cold stress without irrigation showed normal vigor, in contrast to WT plants, which wilted. Transcriptome analysis of *vinv* lines revealed upregulation of an osmoprotectant pathway and ethylene‐related genes during cold temperature exposure. Accordingly, higher expression of antioxidant‐related genes was detected after exposure to short and long cold storage. Sugar measurements showed an elevation of an alternative pathway in the absence of VInv activity, raising the raffinose pathway with increasing levels of myo‐inositol content as a cold tolerance response.

## Significance Statement

Genome editing was used to discover alternative plant tissue pathways for coping with cold stress. Knockout of sucrose degradation gene increased tuber antioxidant defense and enhanced the osmoprotection pathway through the elevation of myo‐inositol levels.

## INTRODUCTION

Cold stress is one of the most devastating abiotic stresses. It impairs plant growth and development, reduces their productivity, and limits their geographical distribution. Reactive oxygen species (ROS) accumulate under cold stress due to a disrupted balance between ROS production and ROS scavenging (Miller et al., 2010; Mittler, 2002). ROS oxidize lipids, proteins, and nucleic acids, which activate further stress responses, leading to cell membrane damage and imbalanced osmotic potential (Cabello et al., 2014; Theocharis et al., 2012).

Plants' survival depends on their response to changes in growth conditions, the severity and duration of stress conditions, and the capacity to quickly adapt to changing energy equations (Miller et al., 2010). Plants cope with cold stress by differentially regulating many genes at the transcriptional level (reviewed by Zhang et al., 2019). These cold‐responsive genes have a role in stress signal transduction and gene expression regulation; their products protect plant cells against damage derived from stresses and maintain cell viability (Yamaguchi‐Shinozaki & Shinozaki, 2005). Plant defense against cold stress is composed of two primary response mechanisms: (i) enzymatic components and (ii) non‐enzymatic antioxidants (Das & Roychoudhury, 2014).

Modulating the expression of genes related to the production and accumulation of compatible solutes helps plants tolerate osmotic stress by maintaining water potential and protecting cellular organelles and essential proteins (Khan et al., 2015). Soluble sugars are important osmoprotectants that play a large role in cellular osmotic adjustment by protecting cell structures that are exposed to environmental stress (Liu et al., 2015; Qi et al., 2007). Other essential water‐soluble carbohydrates derived from sucrose include the raffinose family oligosaccharides (RFOs: α‐galactosyl extensions of sucrose). Sucrosyl oligosaccharides and the enzymes associated with their metabolism might interact indirectly with ROS signaling pathways (Bolouri‐Moghaddam et al., 2010). In addition, RFOs and galactinol have been proposed to have important roles in oxidative stress protection in plants during cold acclimation (ElSayed et al., 2014; Nishizawa et al., 2008; Valluru & Van den Ende, 2008). Therefore, the production of sugars is beneficial for plant survival during cold stress.

Potato (*Solanum tuberosum* L.) is the third most important food crop in the world (after rice and wheat), feeding more than a billion people worldwide (http://cipotato.org/potato). Post‐harvest potato tubers must be stored at cold temperature to prevent sprouting and minimize losses to disease, so as to supply consumers and the processing industry with high‐quality tubers year‐round (Hou et al., 2017). Cold storage triggers cold‐induced sweetening (CIS), characterized by the accumulation of hexoses, such as glucose and fructose, in the tuber parenchyma (Sowokinos, 2001). Heat processing causes hexose to react with free amino acids (e.g., asparagine) via the non‐enzymatic Maillard reaction, producing an unsatisfactory dark color with a bitter‐tasting product and forming the cancer‐causing agent acrylamide (Tareke et al., 2002). Therefore, the identification and development of potatoes that are resistant to CIS are of high priority (Bhaskar et al., 2010; Zhu et al., 2014). Sucrose is cleaved to hexoses by two main enzymes: sucrose synthase (EC 2.4.1.13) and invertase (EC 3.2.1.26). Sucrose synthase catalyzes the reversible conversion of sucrose to uridine diphosphate‐glucose and fructose, and invertase irreversibly splits sucrose into fructose and glucose (reviewed by Koch, 2004).

Sucrose hydrolysis by vacuolar acid invertase (VInv) has been reported to be the main pathway involved in potato CIS (Lin et al., 2015, Zhang et al., 2014, Zhu et al., 2014). Accordingly, *VInv* transcription is upregulated in cold storage and is associated with CIS formation (Bhaskar et al., 2010). RNA interference (RNAi) suppression of *VInv* expression decreased CIS in all cultivars tested (Wiberley‐Bradford et al., 2014, Ye et al., 2010, Zhu et al., 2014). Effective knockout of *VInv* using transcription activator‐like effector nuclease (TALEN) reduced the accumulation of hexoses (Clasen et al., 2016), and overexpression of an invertase inhibitor from tobacco (*Nicotiana benthamiana*) decreased CIS symptoms (Greiner et al., 1999).

In the present study, we induced mutations in the gene encoding VInv in potato tubers of tetraploid cultivars using the CRISPR/Cas9 system, by stable *Agrobacterium* transformation or transient expression in potato protoplasts. Mutations in a few of the alleles dramatically reduced VInv activity and the content of hexoses produced in cold‐stored tubers, similar to a mutation in all four alleles. Transcriptome analysis revealed that *VInv*‐knockout lines exposed to cold stress differentially upregulate ROS scavenger‐encoding genes and an osmoprotectant pathway, leading to an enhanced ROS detoxification phenotype.

## RESULTS

### Generation of *VInv* ‐knockout lines

**Figure 1:**
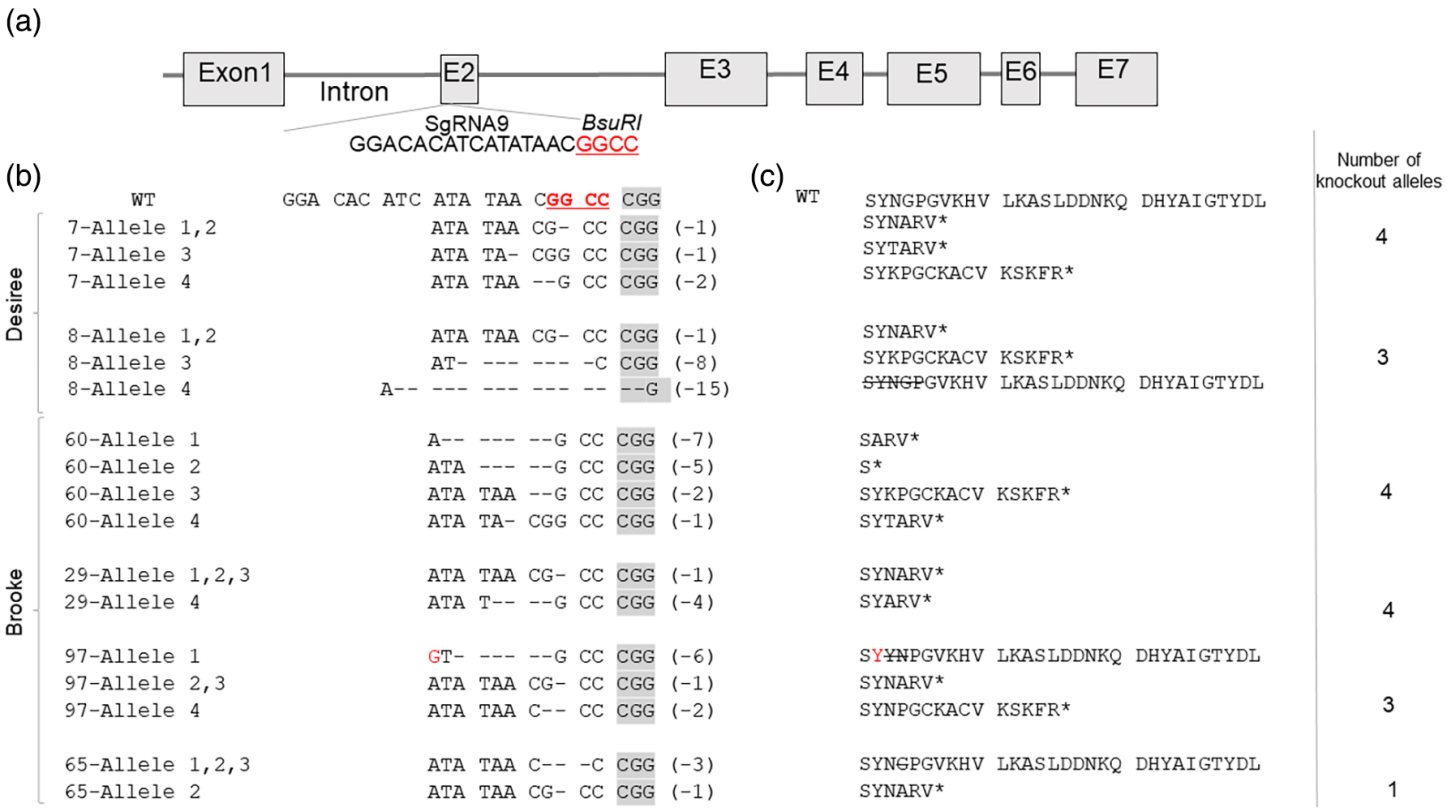
Genotyping of *VInv‐*knockout lines generated in potato cvs. ‘Brooke’ and ‘Désirée’. (a) Schematic presentation of the *VInv* gene and the sgRNA9 target site. Specific restriction sites for the identification of mutated alleles are underlined and marked in red. (b) Sequence analysis of four types of alleles. The WT sequence is shown at the top of each alignment, with the protospacer adjacent motif (gray) and the *Bsu*RI restriction site (bold and underlined in red). Dashes show DNA deletions, and deletion sizes (number of missing nucleotides) are marked on the right side of the sequence. (c) Predicted polypeptides from *vinv* mutant lines. The asterisk marks a predicted stop codon, deleted amino acids are crossed out, and amino acid replacement is shown in red font.

We hypothesized that inducing mutations in all four potato alleles using the CRISPR/Cas9 system would be highly efficient at preventing CIS symptoms. To design the required single guide RNA (sgRNA), the *VInv* gene was analyzed in the reference potato genome *S. tuberosum* Group Phureja clone DM1‐3516R44 (The Potato Genome Sequencing Consortium, 2011). This analysis revealed that *VInv* (PGSC0003DMG400013856) is located on chromosome 3, containing seven exons and six introns (Figure 1a). The sgRNA sequence sgRNA9 was selected for Cas9‐mediated mutagenesis based on homology to potato cvs. ‘Désirée’ and ‘Brooke’. The sgRNA target site was located in exon 2 (Figure 1a). The selected site contains a unique *Bsu*RI restriction site, used to scan for mutant shoots after agrotransformation (Figure 1a) (Salam et al., 2021). Following agrotransformation of cv. ‘Désirée’ with sgRNA9, two *vinv* mutant lines (*#7* and *#8*) were detected following digestion of the relevant PCR product by *Bsu*RI (Figure S1a).

Protoplasts extracted from cv. ‘Brooke’ were transfected with a non‐binary plasmid containing sgRNA9 in the pSAT‐Cas9‐sgRNA (9) plasmid. A regeneration process for protoplasts to shoots was developed in ‘Brooke’ by modifying the method of Nicolia et al. (2015) (Figure S2).

Following genotyping, we found that ‘Désirée’ *VInv*‐knockout line *vinv#7* contained four mutated alleles resulting in a stop codon. The mutant *vinv#8* contained three mutated alleles resulting in a stop codon and a 15‐bp deletion in the fourth allele (Figure 1b,c; Salam et al., 2021). In ‘Brooke’, four mutants (*vinv#29*, *#60*, *#65*, and *#97*) were found (Figure S1b); lines *#60* and *#29* contained four alleles with stop codons, while lines *#97* and *#65* contained three stop codons and one stop codon, respectively (Figure 1b,c; Figure S1c–e). The ‘Brooke’ lines were analyzed for non‐intentional transgenic residues in the genome by using specific primers for the Cas9 protein and sgRNA, showing that only one line (*#65*) was transgenic (Figure S1f,g).

### Knockout of *VInv* decreases CIS even if only some of the alleles are mutated

**Figure 2:**
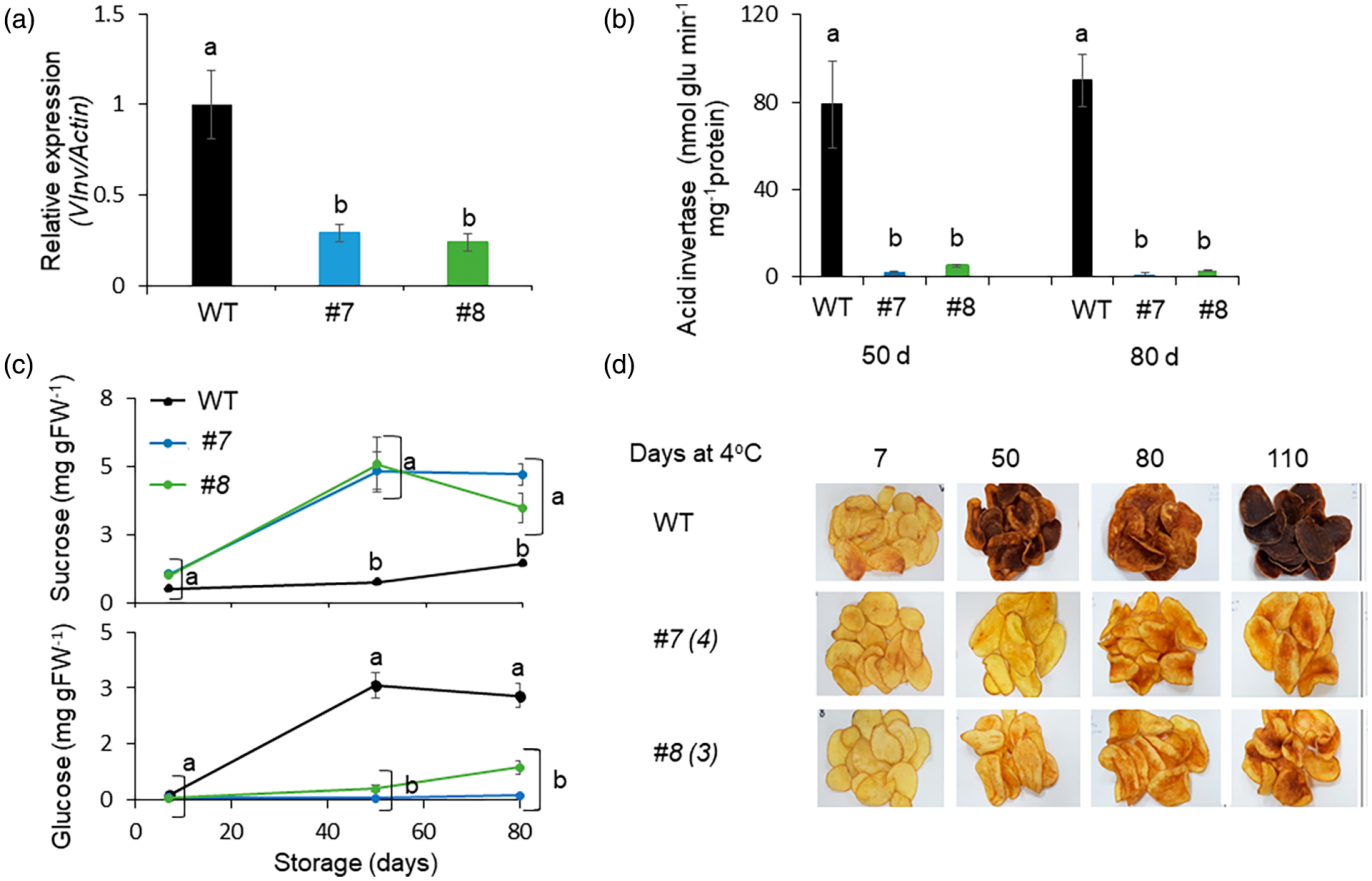
Transgenic knockout of the *VInv* gene reduces CIS in ‘Désirée’ tubers. (a) Expression of *VInv* in WT and *vinv* lines (*#7* and *#8*) after 7 days of cold storage (4°C) as determined by quantitative RT‐PCR analysis using *Actin* as the reference gene. (b) Analysis of VInv activity in *vinv* mutant lines after 50 and 80 days of cold storage (4°C). (c) Sucrose and glucose content in *vinv* lines stored for 80 days at 4°C. (d) Potato chip colors for lines *#7* and *#8* which have four and three mutated (to stop codon) alleles, respectively (marked in parentheses). Bars represent mean ± SE of five independent tubers. Different letters represent significant differences between genotypes (*P* < 0.005) analyzed by one‐way anova followed by the Tukey–Kramer test.

**Figure 3:**
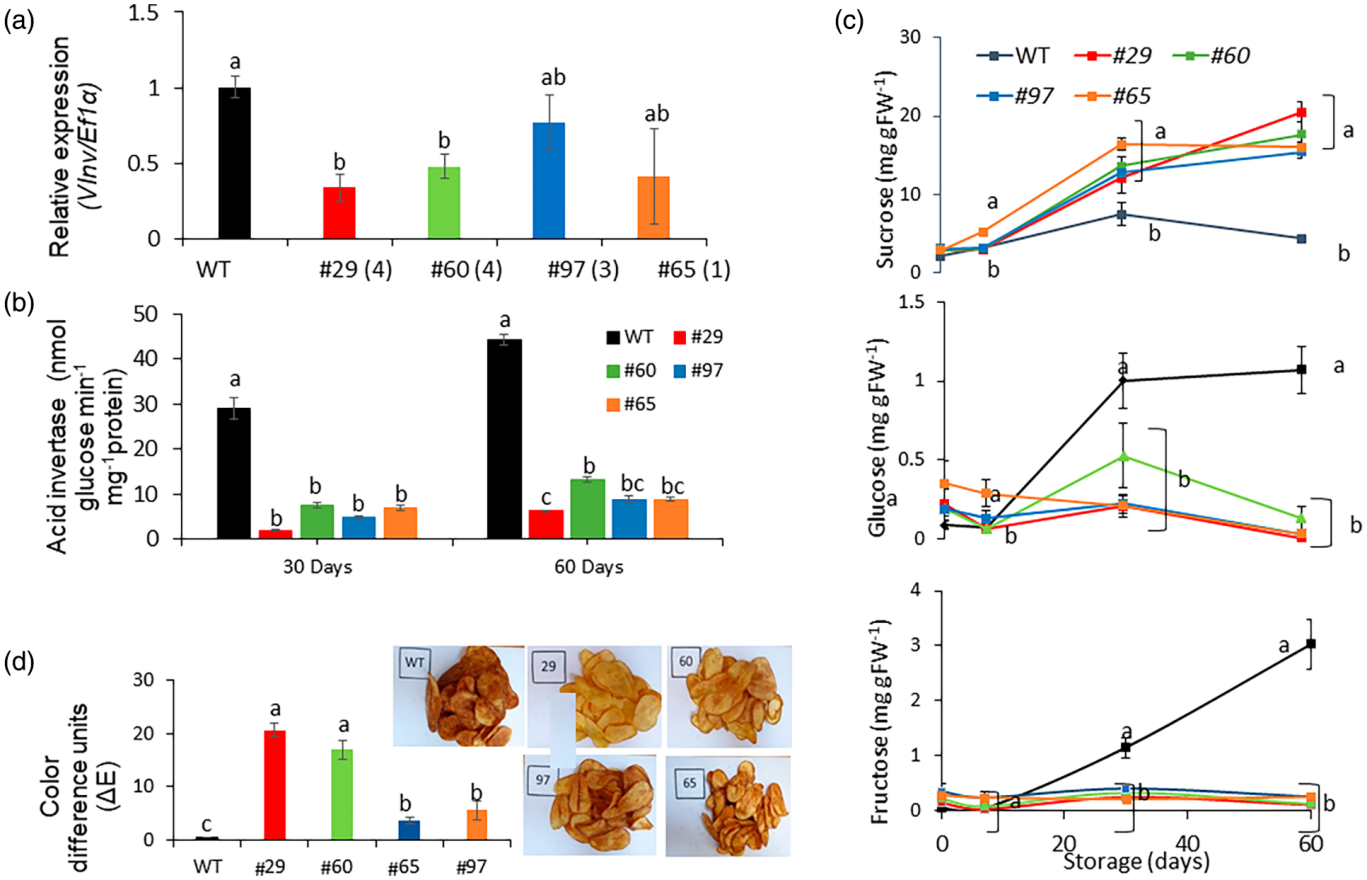
Non‐transgenic knockout of the *VInv* gene reduces CIS in ‘Brooke’ tubers. (a) Quantitative RT‐PCR analysis of *VInv* gene expression in WT compared to *vinv* (lines *#29*, *#60*, *#65*, and *#97*) tubers after 7 days of cold storage (4°C). Lines *#29*/*#60*, *#97*, and *#65* have four, three, and one mutated (to stop codon) alleles, respectively, marked in parentheses. (b) VInv activity after 30 and 60 days of cold storage. (c) Sucrose, glucose, and fructose accumulation during 60 days of cold storage. (d) Colorimetric analysis of tuber parenchyma slices after being fried (Δ*E* units represent color differences between two CIELab colors) and representative images. Bars represent mean ± SE of five independent tuber samples. Different letters represent significant differences between genotypes (*P* < 0.005) analyzed by one‐way anova followed by the Tukey–Kramer test.

To explore the phenotypic consequences of *VInv* mutations, ‘Désirée’ and ‘Brooke’ CRISPR lines were grown under greenhouse conditions for up to 120 days. We did not notice any significant effect of the mutations on overall plant growth, green biomass phenotype, or tuber formation (Figure S3). Interestingly, VInv gene expression and enzymatic activity were lower than in the wild type (WT) in all knockout lines (Figures 2a,b and 3a,b, respectively), which may indicate rapid degradation of non‐functional mRNA. Accordingly, these mutant lines contained higher sucrose levels and lower hexose levels during cold storage (Figures 2c and 3c). After cold storage, fried tuber slices from ‘Désirée’ and ‘Brooke’ mutated lines showed an association between browning and mutation level (Figures 2d and 3d, respectively).

### *VInv* knockout reduces ROS accumulation in response to cold stress

**Figure 4:**
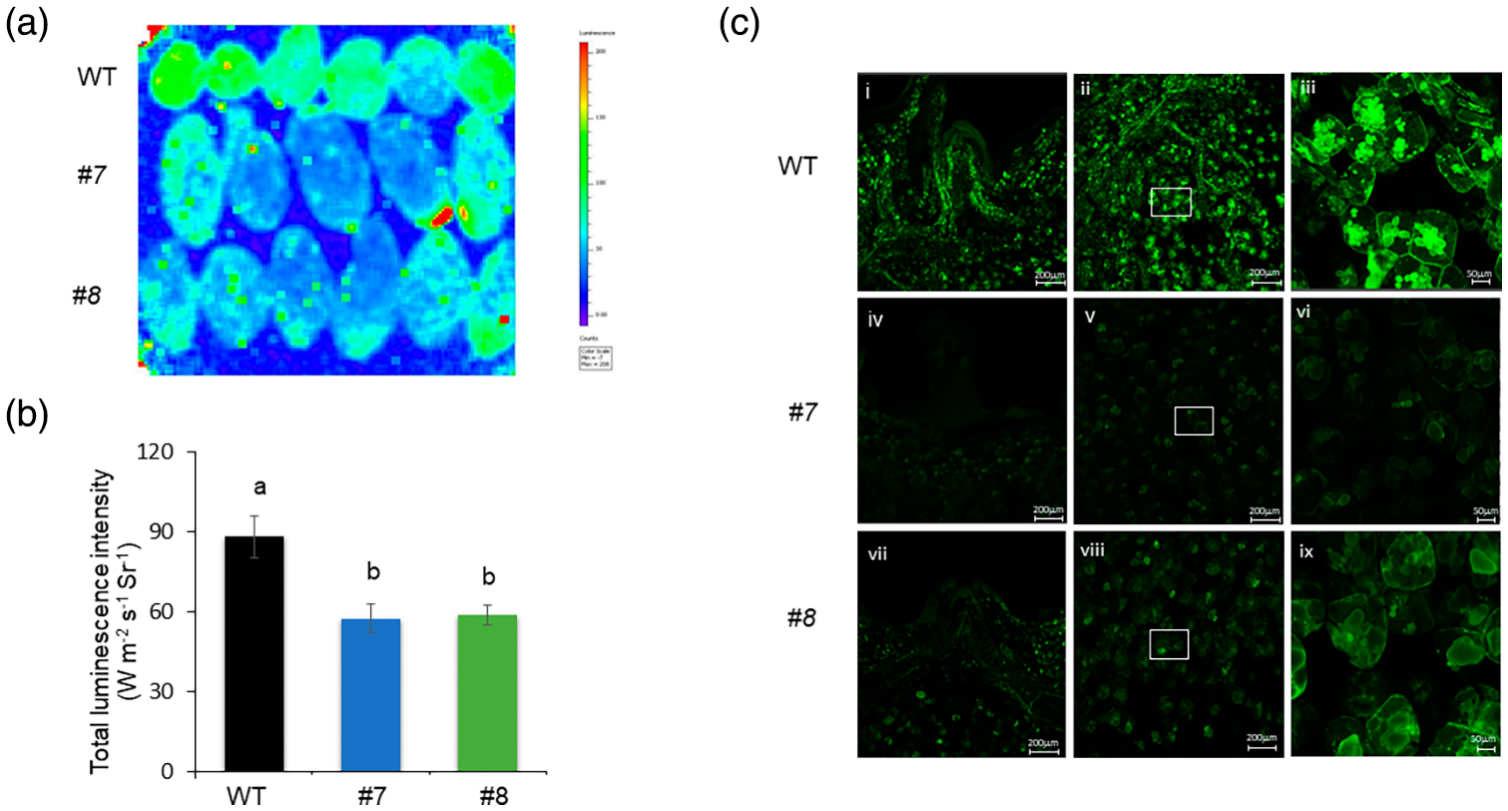
*vinv* mutant lines accumulate lower levels of ROS during cold stress. (a) Representative image of auto‐luminescence of WT and *vinv* mutant lines (*#7* and *#8*) in 10‐day cold‐stored (4°C) tubers using In Vivo Imaging Systems (IVIS). (b) Quantification of total luminescence intensity in terms of radiance (photons sec^−1^ cm^−2^ Sr^−1^) is shown in (a). Bars represent ± SE of three independent repeats (*n* = 10) with 10 tubers per genotype. Different letters represent significant differences between genotypes (*P* < 0.05) analyzed by one‐way anova. (c) Representative fluorescence images of H_2_O_2_ in hand‐cut longitudinal sections of tuber apical bud complex of WT (i–iii), *vinv#7* (iv–vi), and *vinv#8* (vii–ix) stained with BES‐H_2_O_2_‐Ac after 7 days in cold storage. Note the pattern of green fluorescence in correlation with the amount of accumulated H_2_O_2_ in the apical bud (i, iv, vii) and bud‐base parenchyma tissue (ii, v, viii) in WT, *vinv#7*, and *vinv#8*; (iii), (vi), and (ix) are inserts of (ii), (v), and (viii), respectively, showing subcellular H_2_O_2_ localization in the starch granules. Scale bars = 200 μm for (i, ii, iv, v, vii, viii) and 50 μm for (iii, vi, ix).

Since *VInv* knockdown reduces CIS symptoms, we expected *vinv* lines to be less tolerant to cold temperatures. We measured spontaneous photon emission to determine lipid oxidation in tuber parenchyma tissues (Birtic et al., 2011). Surprisingly, *vinv#7* and *vinv#8* showed about 1.5‐fold lower lipid peroxidation levels in response to cold stress (Figure 4a,b). Moreover, they accumulated less H_2_O_2_^−^, as revealed by staining tissues with the fluorescent indicator BES‐H_2_O_2_‐Ac (Maeda et al., 2004). In WT tubers, intense staining of amyloplasts was detected in the bud meristem and the parenchyma located under the bud (Figure 4c). These results suggested that *VInv* knockout enhances the cold protection pathways that reduce ROS accumulation during cold stress. Following these findings, we examined whether whole plants of *vinv* lines can tolerate cold stress better than the WT. Thirty‐two‐day‐old plants were exposed to 2°C for 20 days without irrigation. Mutant plants showed normal vigor, in contrast to the WT plants, which wilted (Figure S4). This experiment suggests that mutant plants cope better with cold stress.

To characterize *vinv* transcriptomes during cold stress, tubers from *vinv#7* and *vinv#8* were exposed to 0, 1, and 50 days of cold storage (4°C), and RNA was extracted from the tuber parenchyma. RNA sequencing (RNA‐seq) analysis resulted in 27 libraries from nine samples (three lines, three time points, and three biological replicates). Each of the cDNA libraries contained 18.5–22.5 million 100‐bp single‐end reads (Table S1). The quality reads of the samples showed 80.4–83.1% mapping to the reference potato genome (Table S1). A Venn diagram was constructed reflecting differentially expressed genes (DEGs) that were upregulated or downregulated following cold treatment to determine the differential gene expression between WT and mutants. Exposure of the tubers to 4°C for 1 or 50 days induced differential expression of 158 (106 upregulated and 52 downregulated) and 772 (212 upregulated and 560 downregulated) genes in *vinv#7* and *vinv#8* compared to the WT, respectively (Figure S5; Table S2). The common groups of upregulated and downregulated DEGs were subjected to Gene Ontology (GO) enrichment analysis using the Blast2GO tool. The most significantly differentially expressed transcripts (log_2_(fold change [FC]) ≥ 2; *P* < 0.05) identified between the WT and both mutants were involved in oxidative stress, carbohydrate metabolism, and ethylene pathways (Figure S6; Table S3).

### Genes related to ROS scavenging and osmoprotection are differentially upregulated under cold stress in *vinv* mutants

**Figure 5:**
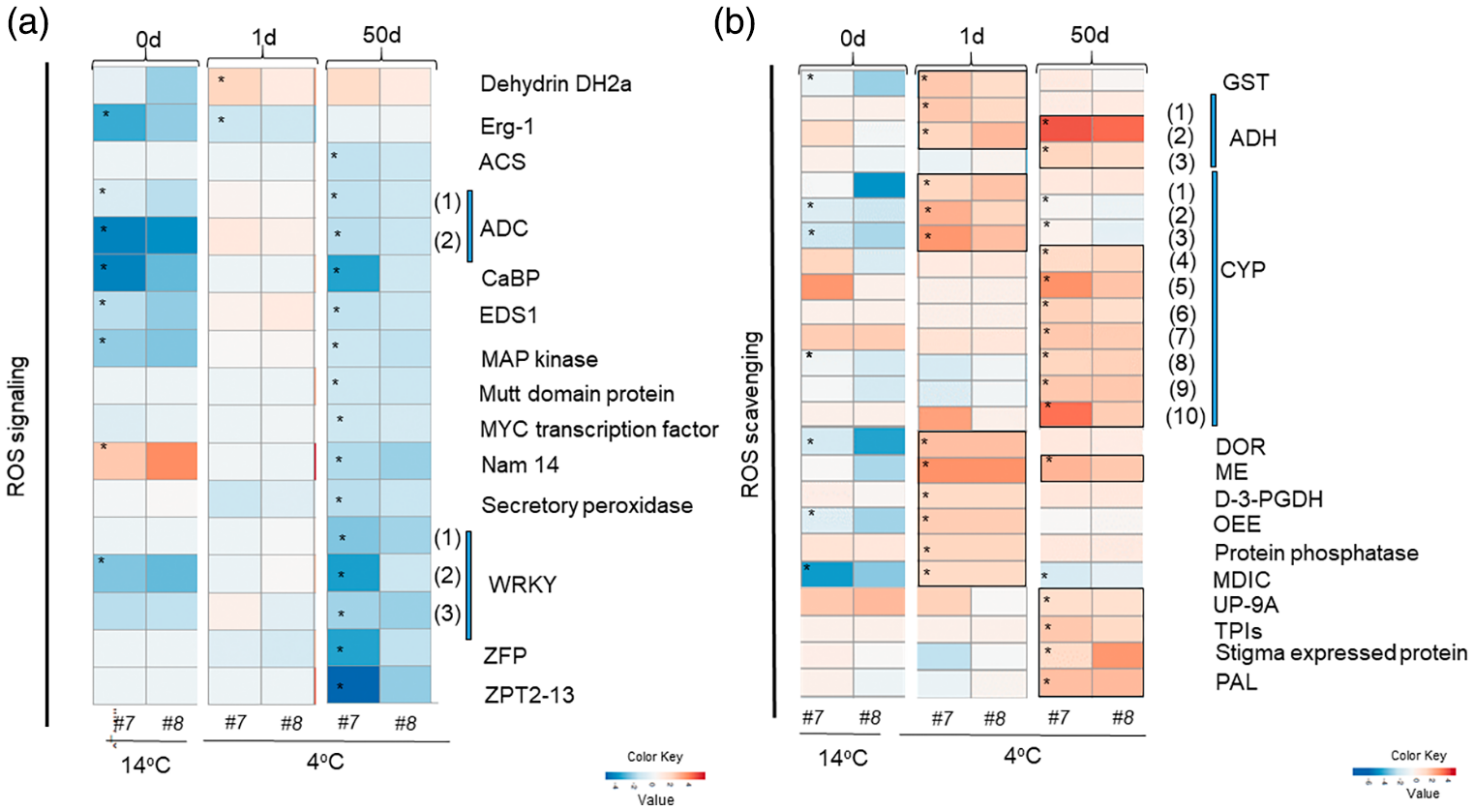
The *vinv* mutant lines display differential ROS responses and scavenging genes during cold stress. Heatmap describing the expression profiles of genes related to (a) ROS signaling and (b) ROS scavenging. Relative expression was quantified by comparing both mutant *vinv#7* (*#7*) and *vinv#8* (*#8*) lines to the WT after 0 (14°C baseline), 1, and 50 days of cold storage (4°C). DEGs are shown according to their expression values (FPKM; average of replications) which were log_2_‐transformed. Expression values are scaled per gene; brown to blue colors indicate higher to lower gene expression, respectively, in lines *#7* and *#8* compared to the WT. Asterisk represents a significant change (*P* < 0.05) in the listed gene. Accession numbers are provided in Table S4a. The numbers in brackets indicate the serial number within the same gene family. Erg‐1, Eca response gene 1; ACS, 1‐aminocyclopropane‐1‐carboxylate synthase; ADC, arginine decarboxylase; CaBP, calcium binding protein; EDS1, enhanced disease susceptibility 1; MAP, mitogen‐activated protein; ZFP, C2H2‐type zinc finger protein; GST, glutathione *S*‐transferase; ADH, alcohol dehydrogenase; CYP, cytochrome P450; DOR, disulfide oxidoreductase; ME, malic enzyme; D‐3‐PGDH, d‐3‐phosphoglycerate dehydrogenase; OEE, oxygen‐evolving enhancer; MDIC, mitochondrial dicarboxylate carrier; TPI, trypsin proteinase inhibitor; PAL, phenylalanine ammonia‐lyase.

We evaluated genes classified to the GO term (GO:0006979) oxidative stress response (Figure 5a; Table S4a). In mutant lines *vinv#8* and *vinv#7*, a pattern of downregulation of ROS signaling was detected. The ROS signaling‐related genes encoding arginine decarboxylase (*ADC1*,*2*), calcium binding protein (*CaBP*), enhanced disease susceptibility protein 1 (*EDS1*), mitogen‐activated protein (MAP) kinase, and *WRKY1–3* were downregulated, suggesting that *vinv* tubers have a significantly higher antioxidant response (Figure 5a). To understand the mechanism that maintains low oxidative stress in *vinv* mutant lines, we analyzed the expression of genes related to ROS scavenging and osmoprotection. During exposure to cold stress, differential upregulation of ROS scavenger‐encoding genes was detected in the *vinv* mutants (Figure 5b; Table S4b). Genes encoding glutathione *S*‐transferase (*GST*), alcohol dehydrogenase (*ADH1*,*2*), cytochrome P450 (*CYP1*–*3*), disulfide oxidoreductases (*DORs*), malic enzymes (*MEs*), and oxygen‐evolving enhancer protein 1 (*OEE*) were upregulated in *vinv* mutant lines after 1 day at 4°C (Figure 5b). Prolonged cold storage of 50 days caused upregulation of *CYP4*–*10*, *ADH2*,*3*, *MEs*, and genes encoding trypsin proteinase inhibitors (*TPIs*) and phenylalanine ammonia‐lyase (*PAL*) in the *vinv* lines.

**Figure 6:**
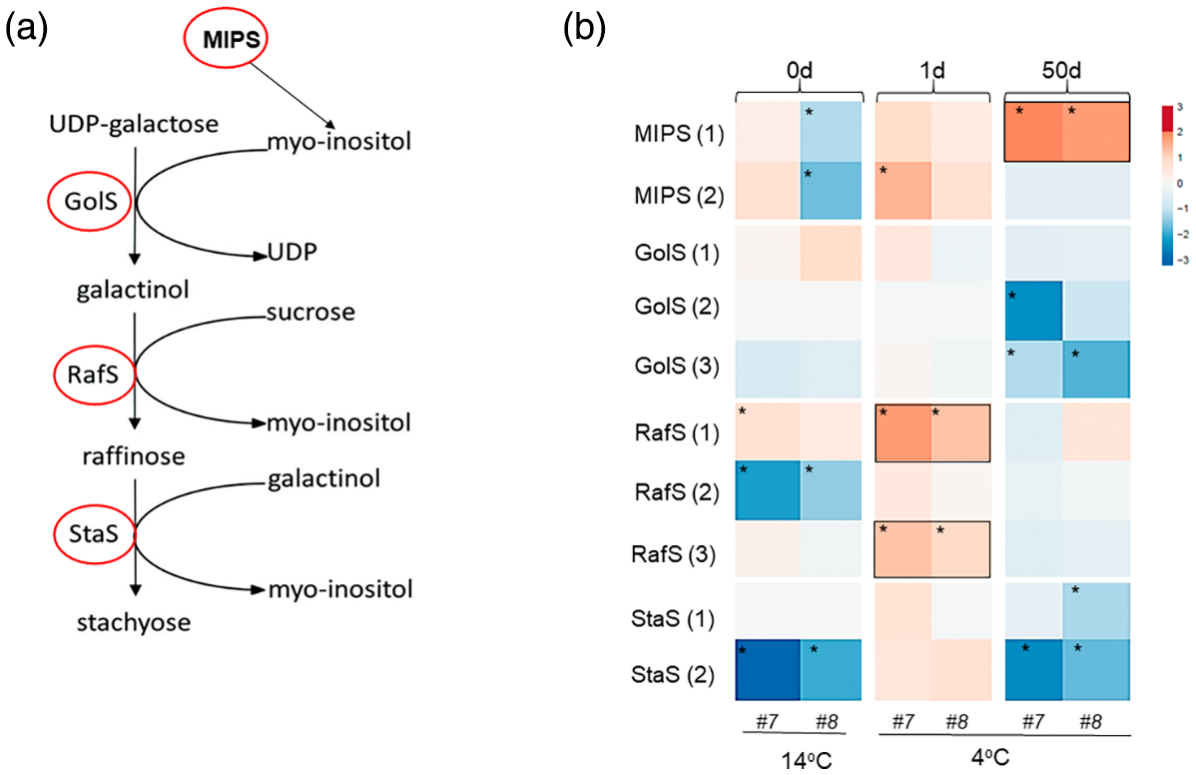
The *vinv* mutant lines display differential expression of stachyose biosynthesis genes following cold stress. (a) Biosynthetic pathway of galactinol, raffinose, and stachyose in plants, adopted from KEGG map ko0052 (figure adopted from Gangl et al., 2015). (b) Heatmap of relative gene expression, quantified by comparing *vinv* lines (*#7* and *#8*) to the WT after 0 (14°C baseline), 1, and 50 days of cold storage (4°C). DEGs are shown according to their expression values (FPKM; average of replications), which were log_2_‐transformed. Expression values are scaled per gene; brown to blue colors indicate higher to lower gene expression, respectively, in lines *#7* and *#8* compared to the WT. Asterisk represents a significant change (*P* < 0.05) in the listed gene. Accession numbers are provided in Table S4c. The numbers in brackets indicate the serial number within the same gene family. MIPS, myo‐inositol‐3‐phosphate synthase; GolS, galactinol synthase; RafS, raffinose synthase; StaS, stachyose synthase.

An important pathway involved in osmoprotection of plant tissue is the synthesis pathway of raffinose and stachyose, which are galactosyl derivatives of sucrose (Peterbauer et al., 2001) (Figure 6a). A single day of exposure to 4°C induced significant upregulation of *RafS1* and *3* (encoding raffinose synthases) in *vinv* lines. After 50 days of exposure to 4°C, the myo‐inositol‐1‐phosphate synthase gene *MIPS1* showed higher expression than in the WT (Figure 6b; Table S4c). This suggests a parallel pathway to cope with cold stress by inducing *RafS* and then *MIPS* expression.

**Figure 7:**
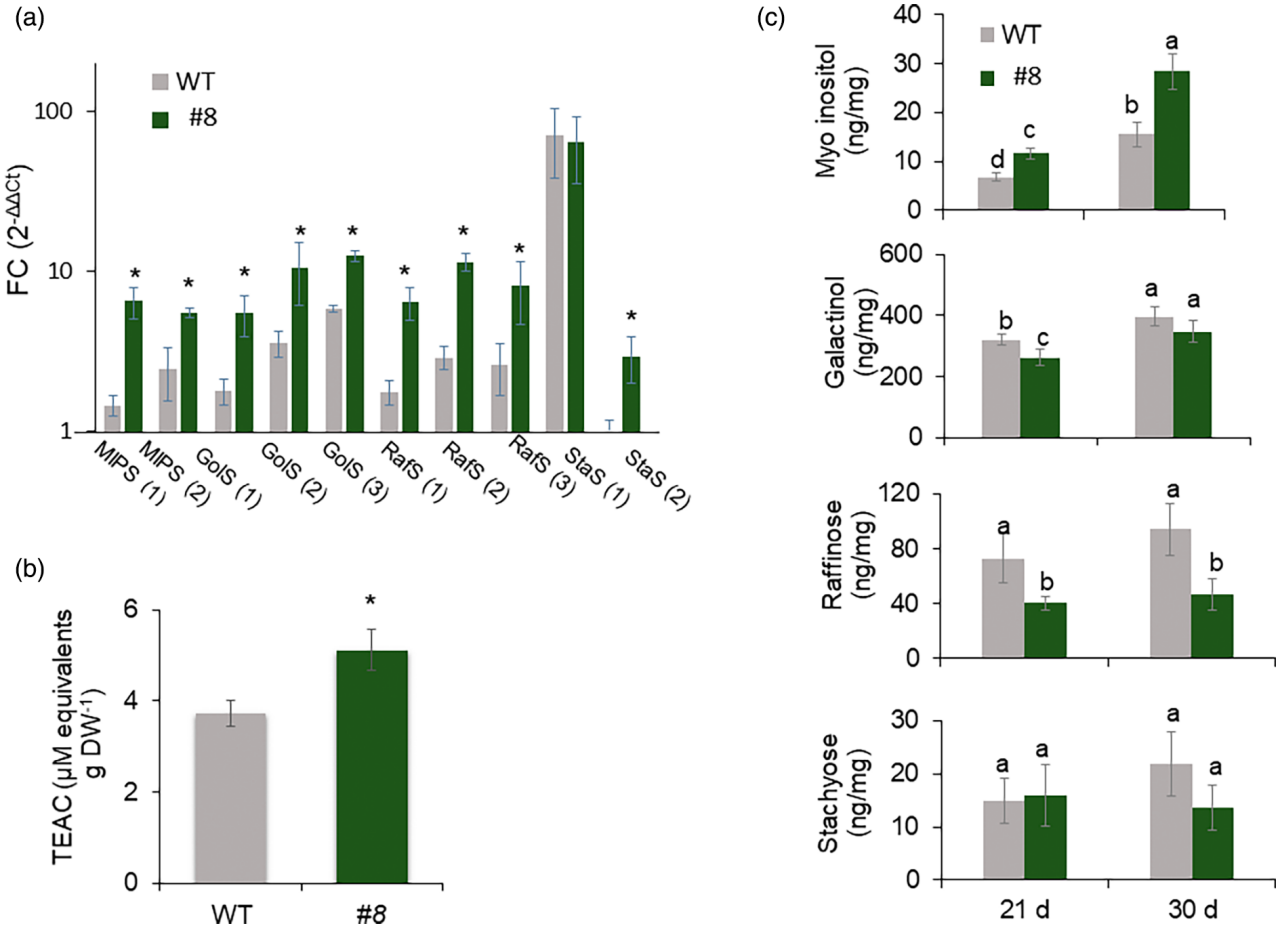
Cold stress induces upregulation of RFO genes in *vinv* mutant lines. Tubers were exposed to cold temperatures (4°C) for 7 days (a, b) and for 21 and 30 days (c). (a) qRT‐PCR analysis of the tuber parenchyma of WT versus *vinv* line *#8*. *Elongation Factor 1‐α* (*EF1α*) was used as the internal control to normalize expression levels, and transcript levels of the tested genes were calculated using the 2^−ΔΔCt^ (FC) method. *MIPS*, myo‐inositol‐3‐phosphate synthase; *GolS*, galactinol synthase; *RafS*, raffinose synthase; *StaS*, stachyose synthase. The numbers between brackets indicate the gene number with in the same gene family. (b) TEAC assay of total parenchyma tissue extracts. (c) Quantification of RFO contents in *vinv* line *#8* as compared to the WT. Asterisks or different letters indicate significant differences between treatments (*P* < 0.05).

We analyzed the expression of key genes related to galactose metabolism by quantitative real‐time PCR (qRT‐PCR) analysis. After 7 days at 4°C, *MIPS 1* and *2*, *GolS1–3* (encoding galactinol synthases), *RafS 1–3*, and *StaS2* (encoding stachyose synthase) had significantly higher expression levels in *vinv#8* compared to the WT (Figure 7a). A Trolox equivalent antioxidant capacity (TEAC) assay was performed on parenchyma tissue extracted from tubers stored at 4°C for 7 days. Antioxidant capacity, as determined by scavenging ABTS^•+^ radical cations, was significantly higher in *vinv#8* than in the WT (Figure 7b), demonstrating enhanced ROS scavenging. Following 21 or 30 days of cold stress, we found that myo‐inositol levels were significantly increased and galactinol, raffinose, and stachyose levels were reduced or not changed compared to the WT (Figure 7c).

## DISCUSSION

### Partial knockout of *VInv* alleles results in improved resistance to CIS

Our results demonstrate that *vinv* mutant tubers display tolerance to cold stress even if only some of the alleles are knocked out (Figures 2 and 3). Suppression of VInv protein activity by knockout of various numbers of *VInv* alleles in ‘Désirée’ and ‘Brooke’ effectively prevented CIS (Figures 2 and 3). An association between VInv activity, hexose content, and frying color was demonstrated for most lines containing three or four knocked out alleles (Figures 1, 2, 3). Using the TALEN system, a previous study showed a positive correlation between the number of WT alleles and hexose levels in cv. ‘Ranger Russet’ (Clasen et al., 2016). Moreover, RNAi silencing of *VInv* reduced glucose and fructose accumulation, whereas partial suppression did not affect CIS (Bhaskar et al., 2010, Wu et al., 2011). Contradicting our results, this latter observation suggested that *VInv* has to be completely silenced to obtain CIS resistance. Precise correlations between the number of knocked out alleles and specific cultivars remain to be evaluated.

The *vinv* lines *#29* and *#60* appeared to exhibit tolerance to CIS, even after 60 days of cold storage, with light‐colored fries, an essential feature for the potato chip industry. The Δ*E* value, which indicates the level of color change according to ‘*L*’, ‘*a*’ and ‘*b*’, reflects the sugar content. Acrylamide content is widely accepted to be highly correlated with hexose content, especially that of glucose (reviewed by Vinci et al., 2012). In addition, acrylamide content is correlated to the ‘a’ value and inversely correlated to the ‘*L*’ value in the colorimetric measurement of frying degree (Bethke & Bussan, 2013, Pedreschi et al., 2005).

Despite its large genetic variation, potato breeding has made only small advances in breeding goals due to the complexity of breeding a heterozygous tetraploid crop (Bachem et al., 2019). Therefore, CRISPR/Cas9 genome editing has become an important tool because it minimizes the introduction of undesired genetic modifications (Li et al., 2019), enabling the breeding of desired traits in existing elite cultivars without affecting their agronomic performance (Xu et al., 2019). For commercial use, the regenerated mutants should lack unwanted insertions of foreign DNA. As a result, in *Agrobacterium*‐mediated mutant plants, the transgene must be segregated out. Since commercial potato cultivars are very heterozygous, cultivar characteristics will probably change. We used transient expression of the CRISPR‐Cas9 plasmid to generate transgene‐free knockout mutants from protoplasts. In this case, changes in chromosomal number/structure due to somaclonal variation, which happens during plant regeneration from protoplasts, must be examined (Fossi et al., 2019), even though these clones have dramatically reduced CIS symptoms with no visible changes in other characteristics of the potato cultivar.

### The *vinv* mutant tubers show lower oxidative stress and higher antioxidant gene expression

Following cold stress, *vinv* lines displayed lower ROS accumulation and less oxidative damage than the WT (Figure 4). Knockout lines showed about 1.5‐fold less lipid peroxidation and lower H_2_O_2_^−^ levels in response to cold stress (Figure 4). Low temperatures usually induce oxidative stress, mediated by the overproduction of ROS such as O_2_^−^, H_2_O_2_, and OH^−^ (Airaki et al., 2012, Mittler, 2002). Accordingly, potato tubers have been shown to produce free radicals under low‐temperature conditions (Wismer et al., 1995). Accumulated soluble sugars serve as osmoprotectants, protecting the cells from low‐temperature damage linked to oxidative stress and ROS signaling (ElSayed et al., 2014).

Expression of antioxidant genes (*GST*, *ADHs*, *CYPs*, *DOR*, *ME*, *OEE*, *TPI*, and *PAL*) and antioxidant capacity were significantly higher in the *Vinv*‐knockout tubers, leading to a lower oxidative stress response (Figures 5 and 7). Upregulation of these ROS‐scavenging enzymes has been reported to be involved in plants' cold tolerance (Gharechahi et al., 2014, Hu et al., 2015, Seppänen et al., 2000).

Our results demonstrate that *vinv* mutant tubers contain higher sucrose levels during cold storage (Figures 2 and 3). Sucrose may serve as a protectant against cold stress by acting as a signal or as an osmoprotectant (cryoprotective) molecule (Tarkowski & Van den Ende, 2015, Van den Ende & Valluru, 2009). Studies conducted in vitro have shown that the ID_50_ value of sucrose, requiered to inhibit OH^•-^, is similar to that of the glutathione antioxidant (Nishizawa et al., 2008). In agreement with this, the invertase inhibitor PpINH1 in peach (*Prunus persica*) maintains high sucrose levels, improves membrane stability during cold storage, and enhances resistance to chilling injury (Wang et al., 2013, 2020). This suggests that *vinv* mutants cope with cold stress in a sucrose‐dependent manner.

An association between elevated sucrose levels and antioxidant enzyme activities has been previously suggested (Couée et al., 2006). Furthermore, overexpression of sucrose transporters has been shown to result in upregulation of most ROS scavengers and to enable plants to overcome abiotic stress (Cai et al., 2017). Cao et al. (2014) reported that sucrose pre‐treatment of cucumber (*Cucumis sativus*) seedlings leads to lower levels of O_2_^•−^ and H_2_O_2_ under chilling stress, demonstrating the ROS scavenging capacity of sucrose.

### The *vinv* mutant lines exhibit enhanced osmoprotection under cold stress

The *vinv* mutant tubers displayed upregulation of the RFO pathway after exposure to cold stress (Figures 6 and 7a). Upregulation of *MIP1*, *GolS*, and *RafS* transcripts was detected after 1–7 days of cold stress (Figures 6 and 7a). Analysis of the RFO contents revealed that *vinv* mutants contain higher levels of myo‐inositol and lower or similar levels of galactinol, raffinose, and stachyose (Figure 7c), indicating that RFO accumulation is used for myo‐inositol synthesis (Figure 6a). Raffinose synthase has been demonstrated to catalyze raffinose formation or galactinol hydrolytic activity to produce myo‐inositol, depending on the substrate content (Li et al., 2017, Li, Zhang, et al., 2020, Peterbauer et al., 2002). We suggest that *vinv* knockout induces sucrose accumulation, which triggers the upregulation of genes involved in RFO synthesis, followed by an enzymatic activity that leads to myo‐inositol accumulation (Figure 9). As previously shown, during drought stress of maize (*Zea mays*) and Arabidopsis plants, galactinol serves as the substrate for the synthesis of raffinose and myo‐inositol (Li, Zhang, et al., 2020). It is suggested that myo‐inositol may contribute to plant tolerance to cold stress, as reported in other plants (Tan et al., 2013, Wang et al., 2022, Zhuo et al., 2013). The tolerance of *vinv* plants to cold and drought stress (Figure S4) suggests that the whole plant is affected by higher sucrose content, but this should be further examined in greenhouse and field experiments.

RFOs are proposed to play an essential role in protecting plants from oxidative stress as they accumulate under stressful conditions (Gu et al., 2018, Morsy et al., 2007, Nishizawa et al., 2008). Accordingly, inhibition of *VInv* expression in *Arabidopsis thaliana* promoted the accumulation of raffinose, increasing the tolerance of transgenic Arabidopsis to cold conditions (Klotke et al., 2004). As a general rule, rice (*Oryza sativa*) and Arabidopsis do not accumulate large quantities of RFOs in their tissues under optimal conditions. However, under stressed conditions, such as extreme temperatures, RafS accumulation is expected (Gangl & Tenhaken, 2016, Saito & Yoshida, 2011). Although there is evidence of a correlation between GolS activity and RFO contents, the concentrations of the initial substrates myo‐inositol and sucrose were associated with RFO accumulation in seeds (Karner et al., 2004). We found sugar‐responsive *cis*‐elements upstream of *RafS1*, *2*, and *3*, including the sucrose‐responsive element (SURE) and SP8 (Rolland et al., 2006). Low‐temperature‐related (LTR) elements, dehydration‐responsive elements (DREs), and C‐repeat binding factor (CBF) binding sites, also known as DREBs, were also found (Qin et al., 2011). Furthermore, *cis*‐elements related to ROS have been predicted for *RafS*1–3 (Li, Yuan, et al., 2020, Nishiuchi et al., 2004) (Table S5), suggesting that ROS molecules and elevated levels of sucrose in *vinv* mutant lines induce *RafS* expression during cold stress. As a result, expression of genes related to myo‐inositol synthesis was induced to enhance cold tolerance.

### *VInv* knockout affects the ethylene biosynthesis pathway

**Figure 8:**
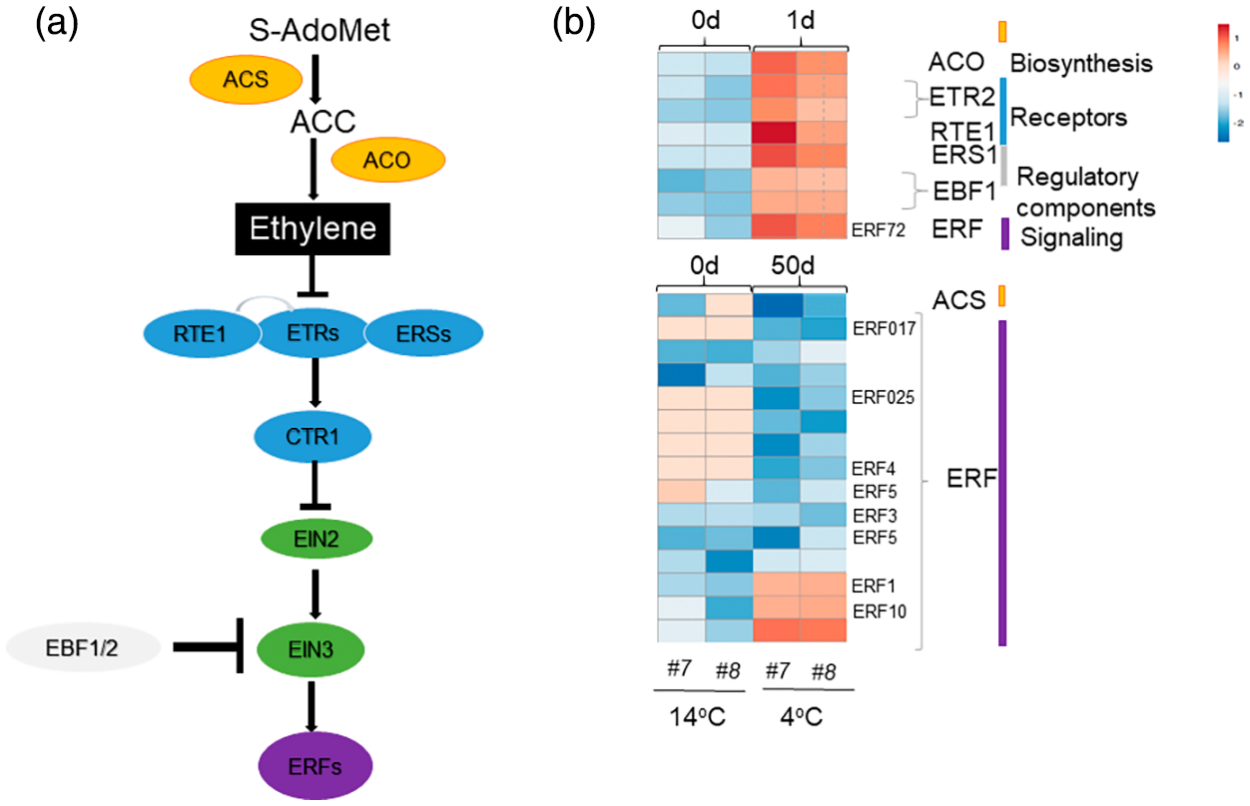
Differential gene expression in the ethylene pathway of *vinv* mutants (*#7* and *#8*) lines during 1 and 50 days of cold storage (4°C). (a) Schematic representation of ethylene biosynthesis, receptors, regulatory components, and signaling molecules. *S*‐Adenosyl methionine (S‐AdoMet) is synthesized from methionine and converted to 1‐aminocyclopropane‐1‐carboxylic acid (ACC) by ACC synthase (ACS). Then, ACC‐oxidase (ACO) catalyzes the release of ethylene from ACC in the presence of oxygen. Perception of ethylene is achieved through ER membrane‐localized receptors (ERSs) and ethylene response sensors (ETRs) that repress ethylene signaling in the absence of ethylene. Receptor activity is modulated by reversion‐to‐ethylene sensitivity1 (RTE1). In the absence of ethylene, ETRs prevent signaling and transcriptional reprogramming by activating the receptor‐associated kinase constitutive triple response 1 (CTR1). CTR1 controls activation of ethylene‐insensitive 2 (EIN2) and EIN3 is degraded by the F‐box proteins EIN3 binding F‐box protein 1 (EBF1) and EBF2 through a 26S proteasome‐mediated degradation pathway. Once ethylene is bound to the receptors, the inhibitory activity of CTR1 is blocked. Release of CTR1 inhibition allows EIN2 to act as a positive regulator of the ethylene signaling pathway. Downstream of EIN3, other transcription factors such as ethylene response factors (ERFs) contribute to transcriptional reprogramming of ethylene‐responsive genes. Proteins are divided into biosynthesis (orange), receptors (blue), and signaling (purple), transcriptional reprogramming (green), and regulatory (gray) components (based on Seyfferth et al., 2018). (b) Heatmap showing the expression profiles of ethylene pathway genes shown in (a). Expression values are scaled per gene; red and blue indicate higher and lower gene expression, respectively, in *#7* or *#8* compared to WT. Accession numbers are provided in Table S4d.

The ethylene biosynthesis pathway was altered in *vinv#7* and *vinv#8* mutants (Figure 8; Table S4d). After 1 day of cold storage, we detected significant upregulation of ethylene biosynthesis‐related genes, receptors, regulatory components, and signaling molecules (Figure 8b; Table S4d). After exposure to 50 days of cold storage, we detected mainly downregulation of ethylene signaling‐related transcripts (Figure 8b). Cold tolerance of Arabidopsis treated with the direct precursor of ethylene 1‐aminocyclopropane‐1‐carboxylate (ACC) is enhanced in soil‐grown seedlings but reduced *in vitro*, and cold tolerance is increased when aminoethoxyvinylglycine (AVG), an inhibitor of ethylene synthesis, is applied (Catalá et al., 2014, Shi et al., 2012). In potato, overexpression of ethylene‐responsive element‐binding protein 1 (StEREBP1) induces the expression of several GCC‐box‐containing stress response genes and enhances tolerance to cold (Lee et al., 2007). The induction of the ethylene biosynthesis pathway by cold stress in potato tubers and its relation to sugars warrants further investigation.

**Figure 9:**
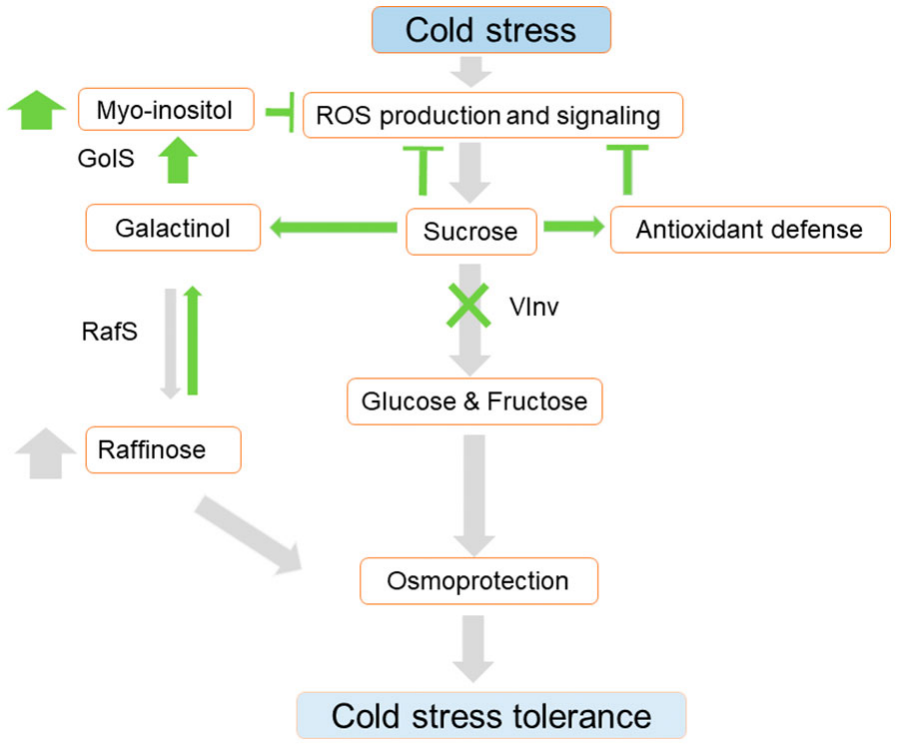
Proposed model of the response of potato tubers to cold stress. During cold stress, sucrose accumulates, enhancing vacuolar invertase (VInv) activity. Raffinose synthase (RafS) is upregulated in response, increasing raffinose synthesis (gray arrows). In the absence of VInv activity, sucrose accumulates, increasing galactinol synthase (GolS) activity to produce myo‐inositol (green arrows). Sucrose accumulation enhances the expression of antioxidant‐related genes as well. Both the osmoprotectant and the antioxidant pathway contribute to the enhanced cold tolerance phenotype, represented by low ROS production and signaling in *vinv* mutant lines.

In conclusion, our results suggest that partial or full knockout of *VInv* enhances sucrose accumulation, increases tuber antioxidant defense, and enhances myo‐inositol osmoprotection. The RFO metabolic pathway is affected by the relative quantity of its substrate, sucrose, resulting in either the synthesis of raffinose or the hydrolysis of galactinol to produce myo‐inositol (Figure 9). Non‐transgenic knockout of *VInv* by CRISPR/Cas9 may serve as a way to develop potato cultivars with tolerance to cold stress, an important feature for field and post‐harvest storage.

## EXPERIMENTAL PROCEDURES

### Plant material and sample collection

Potato cvs. ‘Désirée’ and ‘Brooke’ were propagated *in vitro* every 6–8 weeks in 1× Murashige and Skoog (MS) medium (pH 5.8) containing vitamins (Duchefa, Haarlem, the Netherlands), 3% (w/v) sucrose, 0.8% (w/v) Phyto agar (Duchefa), and 8 μm silver thiosulfate. Plants were cultivated in a growth chamber at 25°C with a 16 h light/8 h dark photoperiod. Tubers were produced in pots by transferring *in vitro* grown plants to the greenhouse. Watering was stopped 2 weeks before the tubers were harvested. After harvest, the tubers were incubated for 14 days at 14°C at 95% relative humidity for curing. Then they were transferred to the specified temperature treatments. Samples were taken from the tuber parenchyma under the apical bud using a cork borer (diameter 1 cm, 3 cm penetration), immediately frozen in liquid N_2_, and transferred to −80°C. Samples of 0.25, 1, and 1.5 g were taken for RNA analysis, protein extraction, and sugar analysis, respectively.

For cold stress treatment of whole plants, WT, *vinv#7*, and *vinv#8* potato tubers were planted and grown under a photoperiod of 16 h light/8 h dark (18°C) for 32 days. Plants were irrigated to saturation before being subjected to cold stress (2°C) for 20 days with no irrigation.

### *VInv* sgRNA design and cloning

The sgRNA9 target regions in exon 2 of *VInv* (PGSC0003DMG400013856) were identified using a publicly available CRISPR design web‐based tool (http://crispr.hzau.edu.cn/CRISPR2/). The target sequence, known as the protospacer adjacent motif (*VInv*‐sgRNA9: GGACACATCATATAACGGCC), was located upstream of the NGG trinucleotide (Figure 1). Cloning was performed as previously reported (Salam et al., 2021). Briefly, the *VInv* target sequence, together with the gRNA scaffold, was amplified using two primers (Table S6) and pRCS‐35S:Cas9‐AtU6:sgRNA as the template (Chandrasekaran et al., 2016). The forward primer contained a *Sal*I restriction site as part of the Arabidopsis U6 (AtU6) promoter and the *VInv*‐sgRNA9 target site. The reverse primer of the Pol III terminator sequence contained a *Hin*dIII site. The amplified DNA (138 bp) was cloned into the *Sal*I and *Hin*dIII sites of the pRCS‐35S:Cas9‐AtU6:sgRNA binary plasmid (Salam et al., 2021). The obtained construct was confirmed by sequencing.

Cloning of the non‐binary plasmid, pSAT‐Cas9‐sgRNA (9), was done using primers flanking the AtU6 promotor‐sgRNA9‐gRNA scaffold containing *Age*I restriction sites (Table S6).

### *Agrobacterium*‐mediated transformation

Potato leaves (cv. ‘Désirée’) were used for *Agrobacterium*‐mediated leaf disk infection as described previously (Horsch et al., 1985, Rocha‐Sosa et al., 1989). Transgenic plants were selected on 50 mg L^−1^ kanamycin (Duchefa). Well‐rooted plants were transferred to soil and grown at 25°C in a greenhouse. After 100 days, tubers were harvested and stored at 14°C for 2 weeks prior to exposure to 4°C cold stress.

### Protoplast extraction, transfection, and regeneration

Protoplasts were isolated from potato leaves of cv. ‘Brooke’ as described previously (Andersson et al., 2018), with some modifications. Briefly, 6‐week‐old potato plants were incubated for 24 h in the dark before protoplast isolation. Upper young green leaves from healthy shoots were excised under sterile conditions and incubated with an enzyme solution for cell wall digestion (Table S7). After 14 h of incubation at 26°C in the dark, the solution was filtered using a 70‐μm cell strainer (CELLTREAT Scientific, Pepperell, MA, USA). The filtrate was centrifuged at 4°C at 50 ***g***. The supernatant was removed and mixed with W5 washing solution three times until a transparent solution was obtained (Table S7). After extraction, the quality of the protoplasts was checked under a light microscope, followed by quantification using a hemocytometer. Protoplasts were transfected at room temperature using 40% (w/v) polyethylene glycol, 1500K protoplasts (100 μl), and 5 μg of the vector DNA pSAT‐Cas9‐sgRNA (9). Reactions were stopped after 3 min by incubation in medium E (Nicolia et al., 2015), and the regeneration process was performed according to Andersson et al. (2018).

### Mutant screening and genotyping

Genomic DNA was isolated from T_0_ potato plants by a previously reported method (Dellaporta et al., 1983). The presence of the Cas9 and sgRNA transgene in T_0_ lines was confirmed by PCR using specific primers (Table S6). The transgenic lines were genotyped by searching for mutated sequences using primers flanking the sgRNA of the *VInv* target region (Salam et al., 2021; Table S6). PCR products were digested with the restriction enzyme *Bsu*RI for sgRNA9. The undigested PCR products were purified, cloned into pGEM‐T (Promega, Biological Industries, Kibbutz Beit‐Haemek, Israel), and digested again with *Bsu*RI enzyme for genotype validation as previously described (Salam et al., 2021; Figure S1c). For ‘Désirée’ lines *vinv#7* and *vinv#8*, 20 and 14 colonies were sequenced, respectively (Salam et al., 2021). For ‘Brooke’ lines *vinv#60*, *#29*, *#97*, and *#65*, 7, 11, 12, and 11 colonies were sequenced, respectively (Figure S1d). Both were aligned to the intact *VInv* using the ClustalW BioEdit software program (CopyrightVC 1997–2013; Tom Hall Ibis Biosciences, Carlsbad, CA, USA). According to the ratio obtained between the sequenced colonies, the four‐allele mutation compositions in ‘Désirée’ and ‘Brooke’ were determined.

### RNA extraction and cDNA synthesis

Tissue was ground and RNA was extracted according to Chen et al. (2015), with slight modifications. To the powdered tissue, 800 μl pre‐warmed (65°C) extraction buffer (100 mm Tris–HCl, pH 8.0, 25.0 mm EDTA, 2.0 m NaCl, 3% [w/v] cetyltrimethylammonium bromide, 4% [w/v] polyvinylpyrrolidone 40, and 3% [w/v] β‐mercaptoethanol) was added, followed by incubation for 45 min at 65°C. Chloroform:isoamyl alcohol (24:1, v/v) was added when the mixture cooled to room temperature. The mixture was incubated for 10 min and then centrifuged at 12 400 ***g*** for 20 min at 4°C. The above steps were repeated. RNA was precipitated by the addition of 2 ml LiCl at a final concentration of 3.0 m and incubated for 2 h at −20°C. Following repeated centrifugation, the pellet was washed twice with 2 ml of 70% ethanol, centrifuged for 10 min, and air‐dried at room temperature. Finally, the pellet was resuspended in 1% DEPC‐treated H_2_O. The quality and quantity of the extracted RNA were assessed by a spectrometer (Thermo NanoDrop 2000, Thermo Fisher scientific, Wilmington, NC, USA). DNA was removed by incubating the RNA with DNase (Invitrogen, Carlsbad, CA, USA) for 10 min at 37°C (1 μl DNase for 10 μg RNA). The reaction was stopped by adding DNase inactivation buffer (Invitrogen) and incubating for 5 min at 70°C. cDNA was obtained by reverse transcription performed on 400 ng RNA using reverse transcriptase (PCR Biosystems, Wayne, PA, USA).

### *VInv* expression analysis

qRT‐PCR was performed with SYBR Green mix (Thermo Fisher Scientific, Waltham, MA, USA) using cDNA as a template, with the following program: 2 min at 50°C, 10 min at 95°C, and 40 cycles of 15 sec at 95°C and 60 sec at 60°C. The primers used for qRT‐PCR are presented in Table S6. Fluorescence detection was performed using a Step One Plus Real‐Time PCR system (Applied Biosystems, Foster City, CA, USA). Gene expression was normalized using *Elongation Factor 1‐α* (*EF1α*) or *Actin* expression as an internal control (Nicot et al., 2005). Results were analyzed with Applied Biosystems™ StepOne™ software. Relative expression was calculated using the 2^−ΔΔCt^ method.

### VInv activity analysis

VInv activity was measured as described previously (Miron & Schaffer, 1991), with minor modifications. A parenchyma sample (2 g) obtained from under the apical meristem was pulverized using liquid nitrogen. Then 250 mg was dissolved in 2 ml extraction buffer (25 mm HEPES–NaOH, 7 mm MgCl_2_, 0.5 mm EDTA, 3 mm DTT, and 2 mm diethyldithiocarbamic acid, pH 7.5). After centrifugation at 18 000 ***g*** for 30 min, the supernatant was dialyzed overnight against 25 mm HEPES–NaOH and 0.25 mm EDTA, pH 7.5, and used as a crude extract. VInv activity was measured by incubating 0.3 ml of 0.1 m citrate/phosphate buffer (pH 5.0), 0.1 ml crude extract, and 0.1 ml of 0.1 m sucrose for 16 h at 37°C. The glucose released from the hydrolysis of sucrose was quantified by adding 500 μl Sumner's reagent (3,5‐dinitrosalicylic acid) and immediately heating to 100°C for 10 min to terminate the reaction, followed by chilling at 4°C (Sumner & Graham, 1921). The reduction of dinitrosalicylic acid to 3‐amino‐5‐nitrosalicylic acid by glucose was determined by measuring the absorbance at 550 nm in a spectrophotometer (Amersham Biosciences, Little Chalfont, UK). Quantitation of glucose in each sample was based on glucose standards. VInv activity was expressed as nanomoles glucose formed per milligram protein per minute. The protein concentration was determined in the respective crude extract using Pierce 660 nm Protein Assay Reagent (Thermo Scientific) with BSA as the standard.

### Extraction and quantification of sugars

To analyze sucrose, glucose, and fructose levels, 1 g of tissue was incubated three times in 5 ml of 80% ethanol at 80°C, 45 min each time. The solution was then dried using a speed vacuum (Centrivap concentrator, Labconco, Kansas City, MO, USA), and after dilution in 2 ml ultrapurified deionized water (UPW; Bio‐Lab, Jerusalem, Israel) the sample was passed through a 0.2‐μm membrane filter (Millex‐GV filter unit; Merck Millipore, Tullagreen, Ireland). The filtrate was used for sucrose, glucose, and fructose analyses by ultrafast liquid chromatography (UFLC) in a UFLC system (LC‐10A UFLC 441 series; Shimadzu, Kyoto, Japan) equipped with a SIL‐HT automatic sample injector, pump system, refractive index detector (SPD‐20A), and automatic fraction collector (FRC‐10A). The UFLC system was also equipped with a differential refractometer detector (Waters 410) and an analytical ion‐exchange column (6.5 × 300 nm) (Sugar‐Pak I; Waters, Milford, MA, USA). The mobile phase UPW was eluted through the system for 30 min at a flow rate of 0.5 ml min^−1^, and the column temperature was set to 80°C. The chromatographic peak corresponding to each sugar was identified by comparing the retention time with that of a standard. A calibration curve was prepared using standards to determine the relationship between the peak area and concentration.

Myo‐inositol, raffinose, galactinol, and stachyose were quantified by LC‐MS/MS at the Targeted Metabolomics Unit in the Department of Life Sciences Core Facilities (Weizmann Institute of Science; Rehovot, Israel). Potato disks (150 mg) were frozen, chopped, and powdered in a bead beater. Saccharides were extracted from the powder by shaking with methanol (1 ml) at 70°C for 10 min, with sucralose (10 μl of 10 μg ml^−1^) as an internal standard. Then, the extracts were diluted with water (250 μl) and shaken at 4°C for 10 min. After centrifugation (16 000 ***g***, 4°C, 10 min), the liquid phase was collected and evaporated in a speed vac and then in a lyophilizer. The obtained residues were re‐dissolved in 100 μl of water, centrifuged, and filtered in nano filter vials (0.2‐μm PES; Thomsom Instrument, Oceanside, CA, USA). LC‐MS/MS analysis was performed on a platform consisting of an Acquity I‐class UPLC system and a Xevo TQ‐S triple quadrupole mass spectrometer equipped with an electrospray ion source (Waters). Chromatographic separation was performed on a UPLC BEH Amide column (2.1 × 150 mm, 1.7 μm; Waters) at 45°C using a linear decrease of acetonitrile in 20 mm ammonium carbonate, pH 9.2 from 66.8 to 33.6% during 6 min, with a flow rate of 0.2 ml min^−1^ and an injection volume of 1 μl. The mass spectrometer was operated in negative ion mode, with corresponding multiple reaction monitoring (MRM) parameters: 179 > 161 and 87 *m*/*z* (collision energy 12 and 20 eV, respectively) for myo‐inositol, 341 > 179 and 161 *m*/*z* (collision energy 40 and 30 eV, respectively) for galactinol, 503 > 221 and 179 *m*/*z* (collision energy 34 and 26 eV, respectively) for raffinose, and 394.9 > 358.9 and 396.9 > 360.9 *m*/*z* (collision energy 13 and 15 eV, respectively) for sucralose (IS). The saccharide concentrations were calculated using standard curves with TargetLynx software (Waters).

### Frying test

Tubers from *vinv* mutant and WT lines were fried in a mini fryer (Bartlet Yeoman, Devon, UK) for 3 min at a temperature of 170°C. After frying, the slices were dried on absorbent paper, and color intensity was validated using a CR‐400 Chroma Meter colorimeter (Minolta, Osaka, Japan). The values of ΔL*, Δa*, and Δb* were calculated together to obtain the color difference units (Δ*E*) according to the following formula: $\Delta E_{\mathrm{ab}}^{*}=\sqrt{{\left(L_{2}^{*}-L_{1}^{*}\right)}^{2}+{\left(a_{2}^{*}-a_{1}^{*}\right)}^{2}+{\left(b_{2}^{*}-b_{1}^{*}\right)}^{2}}$, where $X_{1}^{*}$ represents the WT value and $X_{2}^{*}$ represents the sample value.

### Transcriptome analyses

Parenchyma samples were collected from the middle of the tuber of WT ‘Désirée’, *vinv#7*, and *vinv#8* using a cork borer (6 mm) in three biological repeats. Samples were taken after 0 (14°C baseline), 1, and 50 days of cold storage (4°C). The tissue was frozen in liquid nitrogen and stored at −80°C until RNA extraction. Total RNA of each sample was extracted using a Plant RNA Isolation Mini Kit (Agilent, Santa Clara, CA, USA) according to the supplier's instructions. Library preparation and sequencing were performed at Macrogen, Inc. (NGS, Seoul, Korea). Thirty‐three single‐end RNA‐seq libraries with a length of 100 nucleotides were prepared using Illumina Hiseq2000 and Trueseq protocols.

Raw reads were subjected to a cleaning procedure with the FASTX Toolkit (http://hannonlab.cshl.edu/fastx_toolkit/index.html, version 0.0.13.2) as follows: (i) read‐end nucleotides with quality scores of <30 were trimmed using fastq_quality_trimmer; (ii) read pairs were discarded if either one had less than 70% base pairs with a quality score of ≤30 using fastq_quality_filter. Reads obtained after processing and cleaning were mapped to the previously published data PGSC_DM_v4.03_pseudomolecule (Sol Database‐https://solgenomics.net/organism/Solanum_tuberosum/genome) using TopHat v2.1.1 with default parameters (Trapnell et al., 2009).

Differential gene expression analysis was performed using Cufflinks v2.2.1 (Trapnell et al., 2013). This program assembles transcriptomes from RNA‐seq data and quantifies their expression. In the annotation file of *S. tuberosum* Group *Phureja* clone DM1‐3, a total of 39 028 protein‐encoding genes are present. Fragments per kilobase of transcript per million mapped reads (FPKM) values were used to calculate the log_2_(FC) values. Log_2_(FC) values greater than zero indicate upregulation, whereas those less than zero indicate downregulation. A *P*‐value threshold of 0.05 was considered for statistically significant results. Heatmap visualization was performed using R Bioconductor (Gentleman et al., 2004). For Venn diagram construction, we used the ‘Venny’ tool (Oliveros, 2007).

GO and Kyoto Encyclopedia of Genes and Genomes (KEGG) annotations were performed using the GSEA server (https://www.genome.jp/kegg/). GO enrichment analysis was carried out using the Blast2GO (Conesa et al., 2005) program based on Fisher's exact test (Upton, 1992) with multiple testing corrections of the false discovery rate (FDR) (Benjamini & Hochberg, 1995). The threshold was set to an FDR with a corrected *P*‐value of less than 0.05. GO analysis was performed by comparing the GO terms in the test sample to the GO terms in a background reference. GO provides a structured and controlled terminology to define gene products according to three domains: molecular function (the biochemical activity of a gene product), biological process (operations or sets of molecular events to which the gene product contributes), and cellular component (cell parts in which a gene product is active).

### H_2_O_2_ staining and microscopic observation

For H_2_O_2_ detection, approximately 1 cm length × 8 mm width (1 mm thickness) hand‐cut longitudinal sections of tuber apical bud complex were incubated in 20 μm BES‐H_2_O_2_‐Ac (Fujifilm Wako Pure Chemical, Osaka, Japan) for 20 min in the dark. After washing three times with PBS, fluorescent images were taken using an Olympus IX81/FV500 confocal laser‐scanning microscope equipped with a 488‐nm argon‐ion laser and a 405‐nm diode laser, with a detection range of 485–515 nm.

### Evaluation of lipid peroxidation

Oxidation of linolenic acid produces mainly auto‐luminescence at >600 nm, with a major contribution in the wavelength range of 640–695 nm (Birtic et al., 2011). In Vivo Imaging Systems (IVIS; PerkinElmer, Waltham, MA, USA) was used to detect lipid peroxidation. Tubers were pre‐adapted at room temperature (25°C) in the dark for 2 h before evaluation. Lipid peroxidation was detected and visualized by auto‐luminescence of peroxide lipids as described previously (Birtic et al., 2011), using the program sequence setup consisting of auto‐luminescence for 50 min with emission at 640–770 nm and excitation block, binning factor 8, and *f*‐factor 1. The auto‐luminescence was recorded by a highly sensitive charge‐coupled device camera. Optical luminescent image data were displayed in pseudo‐color, which represents intensity in terms of radiance (photons sec^−1^ cm^−2^ Sr^−1^). The measurements were repeated three times with different tubers, and the signal intensity of each optical image was calculated within the regions of interest. The radiance was summed and is presented as average of total flux (W m^−2^ sec^−1^ Sr^−1^) with SE.

### Antioxidant capacity assay

The TEAC assay was conducted as previously reported (Re et al., 1999). Briefly, an ABTS^•+^ radical solution was prepared by mixing 7 μm 2,2′‐azinobis (3‐ethylbenzothiazoline‐6‐sulfonic acid) diammonium salt (ABTS) and potassium persulfate (dipotassium peroxodisulfate) (150 μm) in 0.2 m buffer acetate (pH 4.3). The solution was heated for 60 min at 45°C, protected from light, and stored at room temperature. To check ABTS^•+^ formation, absorbance at 734 nm was determined (absorption had to be between 0.55 and 0.75). As an antioxidant scavenging standard, Trolox (6‐hydroxy‐2,5,7,8‐tetramethychroman‐2‐carboxylic) was used. ABTS^•+^ shows radical‐scavenging ability of antioxidants even when they are present in complex biological mixtures such as plant or food extracts.

To measure antioxidant capacity, parenchyma from under the apical meristem was pulverized using liquid nitrogen. Then 350 mg was dissolved in 1.5 ml of 0.2 m buffer acetate (pH 4.3). After centrifugation at 14 000 ***g*** for 10 min, the supernatant was used as a crude extract. A 40‐μl aliquot of each sample (with three technical repeats) was mixed with 1 ml of the radical solution and incubated for 20 min in the dark. The antioxidants present in the extracts scavenged ABTS^•+^ radical, resulting in decolorization of the mixture proportional to concentration and antioxidant capacity. The decrease in absorption at 734 nm after the addition of sample was used to calculate the TEAC. The antioxidant capacity of the compounds is expressed relative to that of Trolox. TEAC scavenging effects were calculated using the following formula: (Abs sample – Abs blank)/(Abs standard − Abs blank).

### Statistical analysis

Data were analyzed using Microsoft Excel 2010. Analysis of variance (anova) and the Tukey–Kramer test were performed using JMP software (version 3 for Windows; SAS Institute).

## CONFLICT OF INTEREST

The authors declare no conflicts of interest.

## Supporting information


**Figure S1.** Analysis of *VInv*‐knockout lines of cvs. ‘Désirée’ and ‘Brooke’.
**Figure S2.** Protoplast regeneration process for cv. ‘Brooke’.
**Figure S3.** Normal development of *vinv* plants.
**Figure S4.** *vinv* plants exhibit a cold tolerance phenotype.
**Figure S5.** Venn diagram of DEGs between *vinv* mutant lines (*vinv#7* and *vinv#8*) and the WT following cold storage.
**Figure S6.** GO functional classification of DEGs in *vinv#7* and *vinv#8* lines after cold storage.

**Table S1.** Summary of the obtained RNA sequencing data.

**Table S2.** Representative Venn diagram analysis of DEGs in *vinv#7* and *vinv#8* compared to the WT after 1 and 50 days of cold storage.

**Table S3.** GO analysis of DEGs in *vinv#7* and *vinv#8* versus the WT after 1 and 50 days of cold storage. (+) represents the presence of the gene at the relevant GO term.

**Table S4.** Differential expression (log_2_(FC)) of genes related to (a) ROS scavenging, (b) the oxidative response, (c) the galactinol pathway, and (d) ethylene biosynthesis in *vinv#7* and *vinv#8* lines compared to WT after 0, 1, and 50 days of cold storage.

**Table S5.** Sugar‐, low temperature‐, and ROS‐responsive elements in the 2600‐bp 5′‐flanking sequences of *RafS1* (PGSC0003DMG400018109), *RafS*2 (PGSC0003DMG400030891), and *RafS3* (PGSC0003DMG400022258) predicted by PLACE (Higo et al., 1999).

**Table S6.** List of primers used and their purpose.

**Table S7.** Solutions used for protoplast extraction.

## Data Availability

The authors confirm that the data supporting the findings of this study are available within the article and its supplementary materials.
