# Supplementary material for: An alternative pathway to plant cold tolerance in the absence of vacuolar invertase activity
Source: Plant J. 2022 Dec 22;113(2):327–41. doi: 10.1111/tpj.16049 (PMC10107833; doi:10.1111/tpj.16049)
Supplement: Supplementary file 1 — Figure S1. Analysis of VInv‐knockout lines of cvs. ‘Désirée’ and ‘Brooke’. Figure S2. Protoplast regeneration process for cv. ‘Brooke’. Figure S3. Normal development of vinv plants. Figure S4. vinv plants exhibit a cold tolerance phenotype. Figure S5. Venn diagram of DEGs between vinv mutant lines (vinv#7 and vinv#8) and the WT following cold storage. Figure S6. GO functional classification of DEGs in vinv#7 and vinv#8 lines after cold storage. [file TPJ-113-327-s002.pdf]

Supplementary Figures

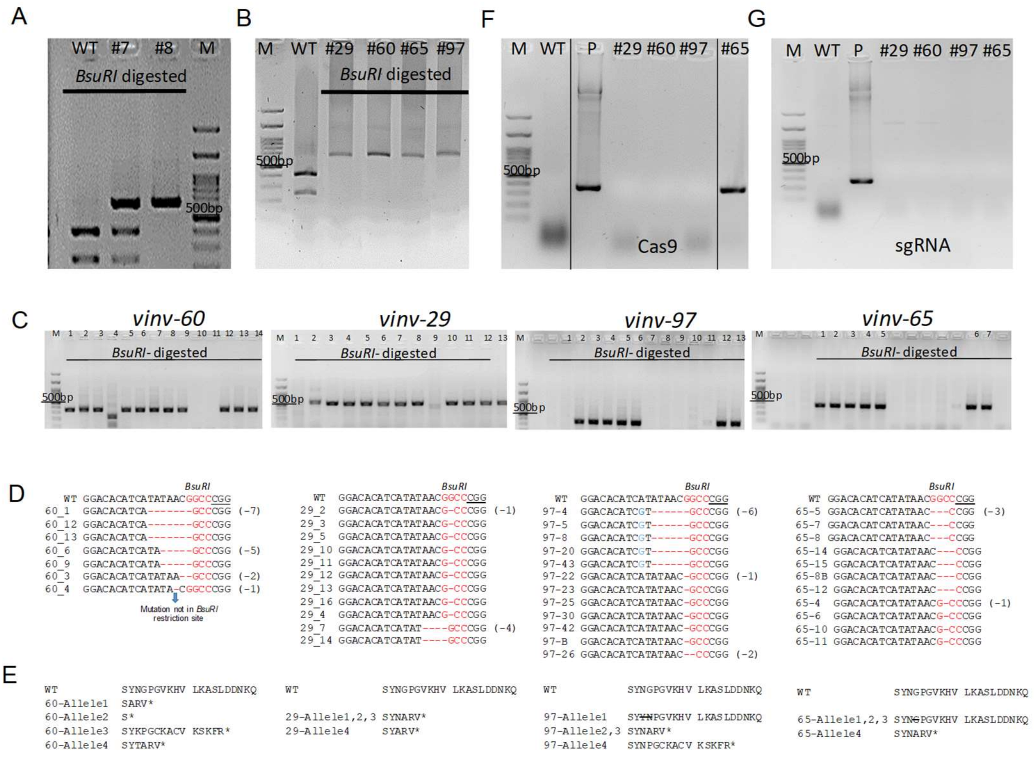

Figure S1. Analysis of *VInv*-knockout lines of cvs. Désirée and Brooke.

Restriction analysis of T_0 PCR fragments of (A) 'Désirée' *vinv* lines *#7, #8* and (B) 'Brooke' *vinv* lines *#29, #60, #65, #97* by *BsurI* restriction enzyme. Uncut band indicates gene editing. Wild type (WT) control shows a cut band. (C) *BsuRI* restriction analysis of Cas9/*VInv*-sgRNA*9* mediated mutations, following colony PCR of T0 PGMT cloned potato plants. PCR was done using a set of primers flanking the sgRNA target site (Salam et al. 2021, Table S6). PCR products that were not digested with the restriction enzyme *BsuRI* were considered mutated. (D) Sequence analysis of colony PCR from representative plants of those shown in (C). WT sequence is shown at the top of each alignment, with the protospacer adjacent motif (PAM; underline) and *BsuRI* restriction site (in red). Dashes indicate DNA deletions, and the number of missing nucleotides is marked on the right side of the sequence, respectively.

Note that in *vinv-60* one colony contains one base pair deletion, not in the restriction site of *BsuRI*, which leads to a stop codon (shown in E). (E) Predicted polypeptides from *VInv* mutant lines. The predicted stop codon is marked by asterisks, and amino acids deleted are erased. (F) Analysis for transgenic Cas9 and **(G)** sgRNA residues in the DNA of lines *#29, #60, #65, #97*. As a control WT and pSAT-Cas9-sgRNA (9) plasmid (P) were used. Lines crossing the gel lengthwise represent places where the gel was cut and reassembled.

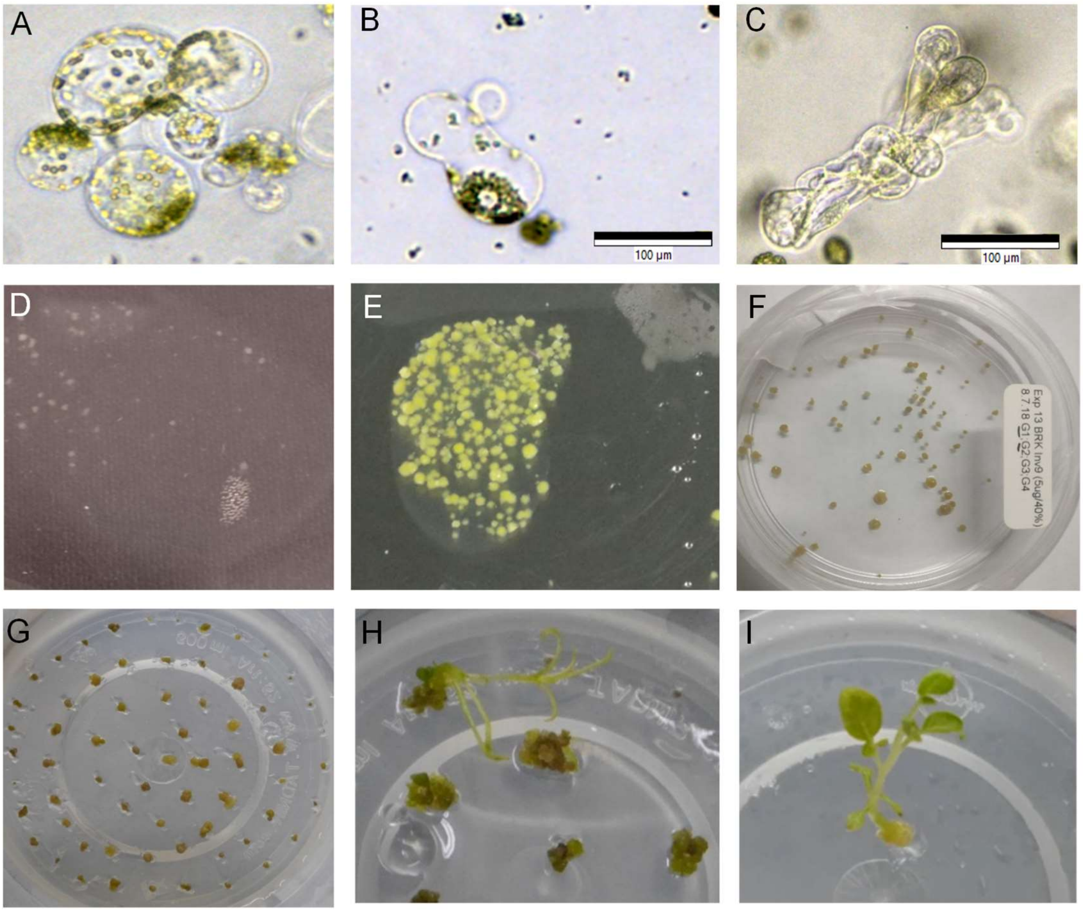

Figure S2. Protoplast-regeneration process for cv. Brooke.

(A) Protoplast extraction. (B) Protoplast division 3 days after extraction. (C) Microcallus 10 days after extraction. (D) At the end of the protoplast extraction, the protoplasts are wrapped in an alginate layer. (E) Callus formation 4 weeks after the extraction. At this point, the callus is released from the alginate and transferred to liquid media. (F, G) Green calluses are transferred to liquid media and later to solid media, respectively. (H, I) Shoot development and transfer to a new solid medium that induces root development, respectively.

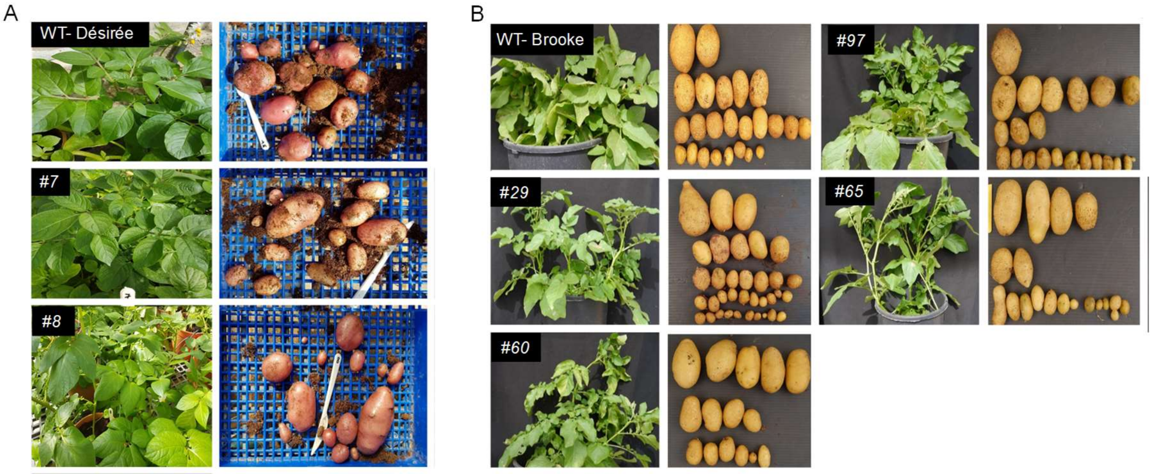

Figure S3. Normal development of *vinv* plants.

(A) ‘Désirée’ WT and *vinv* knockout lines (#7 and #8), and (B) ‘Brooke’ WT and *vinv* lines (#29, #60, #97 and #95) were grown in the greenhouse for 90 and 120 days, respectively.

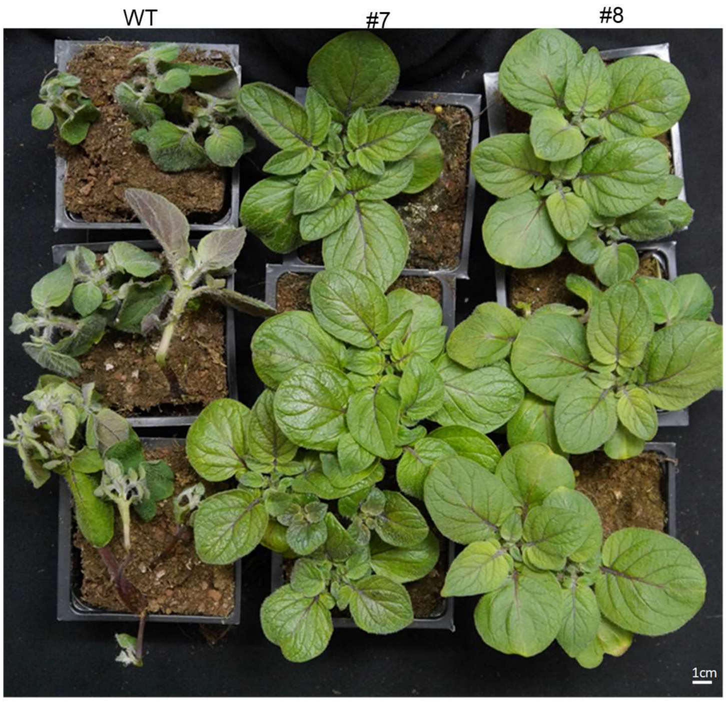

Figure S4*. vinv* lines exhibit cold tolerance phenotype. 32 days-old plants were exposed to cold stress (2°C) for 20 days without irrigation. Wild type (WT) plants suffered from wilting symptoms compared to *vinv* mutants (#7 and #8) that remained with normal turgor.

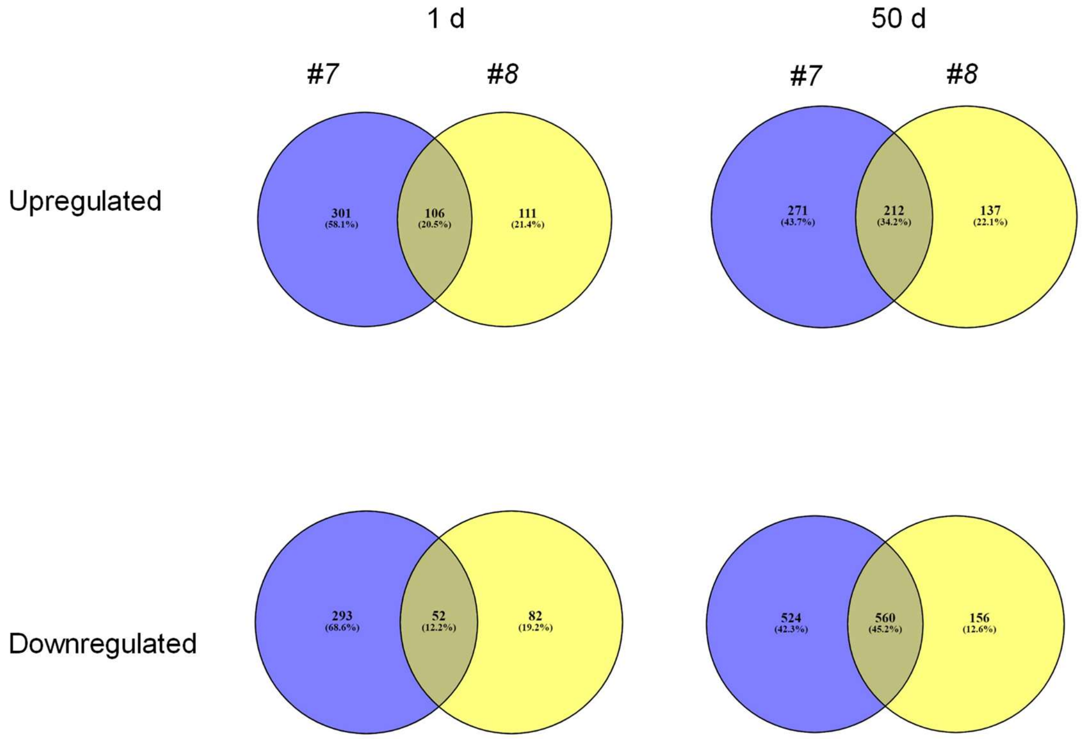

Figure S5. Venn diagram of differentially expressed genes between *vinv* mutant lines (*vinv#7* and *vinv#8*) and the WT, following cold storage (4°C), for 1 (left) and 50 (right) days. Only genes with a 2-fold (log_2) increase or decrease in expression are included.

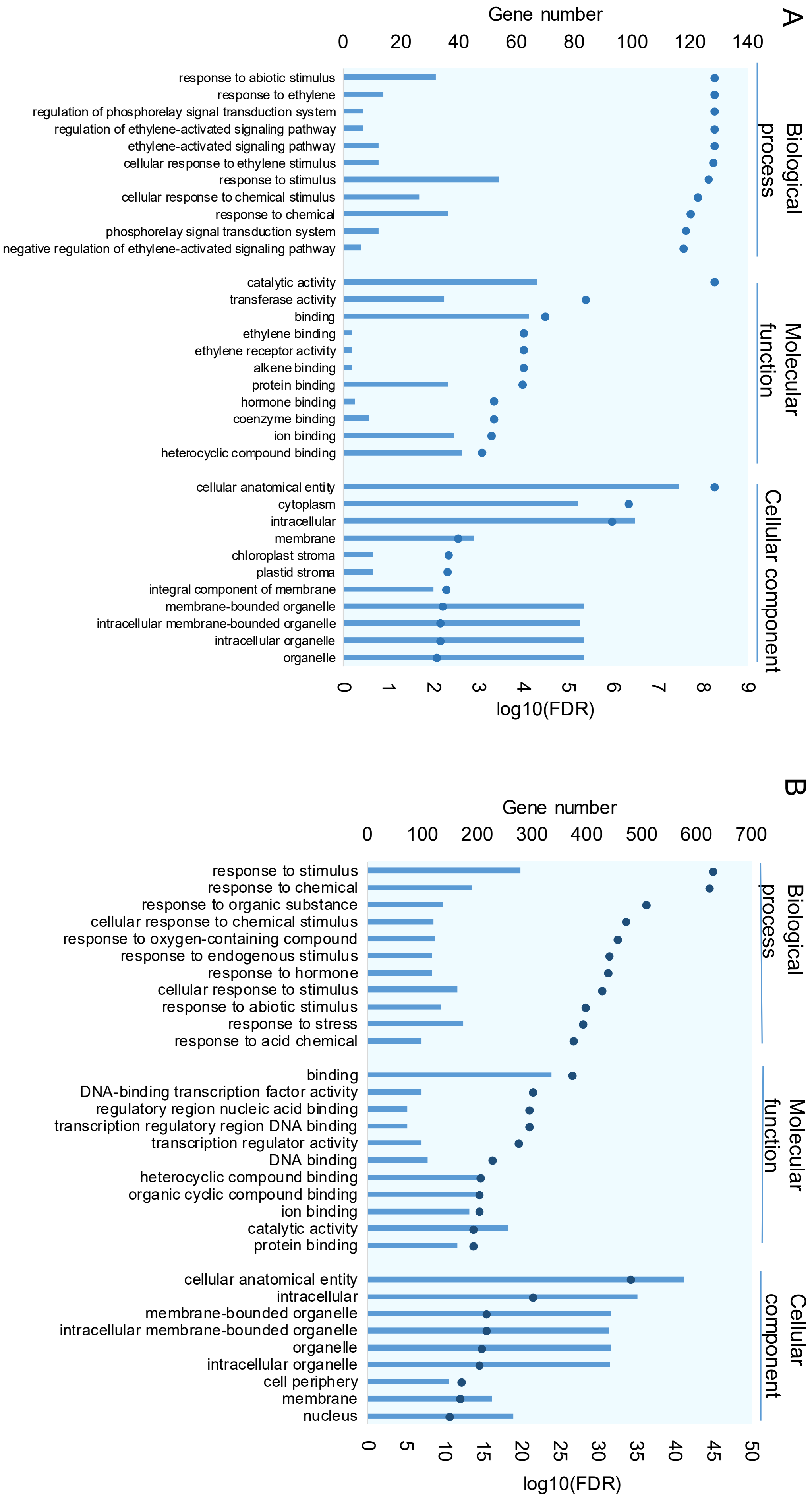
A
Gene number
0
20
40
60
80
100
120
140
response to abiotic stimulus
response to ethylene
regulation of phosphorelay signal transduction system
regulation of ethylene-activated signaling pathway
ethylene-activated signaling pathway
cellular response to ethylene stimulus
response to stimulus
cellular response to chemical stimulus
response to chemical
phosphorelay signal transduction system
negative regulation of ethylene-activated signaling pathway
catalytic activity
transferase activity
binding
ethylene binding
ethylene receptor activity
alkene binding
protein binding
hormone binding
coenzyme binding
ion binding
heterocyclic compound binding
cellular anatomical entity
cytoplasm
intracellular
membrane
chloroplast stroma
plastid stroma
integral component of membrane
membrane-bounded organelle
intracellular membrane-bounded organelle
intracellular organelle
organelle
Biological process
Molecular function
Cellular component
0
1
2
3
4
5
6
7
8
9
log10(FDR)
B
Gene number
0
100
200
300
400
500
600
700
response to stimulus
response to chemical
response to organic substance
cellular response to chemical stimulus
response to oxygen-containing compound
response to endogenous stimulus
response to hormone
cellular response to stimulus
response to abiotic stimulus
response to stress
response to acid chemical
binding
DNA-binding transcription factor activity
regulatory region nucleic acid binding
transcription regulatory region DNA binding
transcription regulator activity
DNA binding
heterocyclic compound binding
organic cyclic compound binding
ion binding
catalytic activity
protein binding
cellular anatomical entity
intracellular
membrane-bounded organelle
intracellular membrane-bounded organelle
organelle
intracellular organelle
cell periphery
membrane
nucleus
Biological process
Molecular function
Cellular component
0
5
10
15
20
25
30
35
40
45
50
log10(FDR)

Figure S6. Gene ontology (GO) functional classifications of differentially expressed genes in *vinv#7* and *vinv#8 lines* after cold storage.

Cold storage for (A) 1 day and (B) 50 days at 4°C. Graphic representation of the top 33 most significantly enriched GO terms classified as cellular component, biological process, and molecular function found for each cluster. The number of genes associated with each GO term is shown as blue bars (left) axes and the log10-transformed *p*-value (FDR) for each GO term is shown as blue dots (right axes). Corrected $P < 0.005$.
