# Supplementary material for: An alternative pathway to plant cold tolerance in the absence of vacuolar invertase activity
Source: Plant J. 2022 Dec 22;113(2):327–41. doi: 10.1111/tpj.16049 (PMC10107833; doi:10.1111/tpj.16049)
Supplement: Supplementary file 6 — Table S5. Sugar‐, low temperature‐, and ROS‐responsive elements in the 2600‐bp 5′‐flanking sequences of RafS1 (PGSC0003DMG400018109), RafS2 (PGSC0003DMG400030891), and RafS3 (PGSC0003DMG400022258) predicted by PLACE (Higo et al., 1999). [file TPJ-113-327-s004.docx]

**Table S5.** Sugar, low temperature and ROS-responsive elements in the 2600-bp 5'-flanking sequence of *RafS1* (PGSC0003DMG400018109), *RafS*2 (PGSC0003DMG400030891) and *RafS3* (PGSC0003DMG400022258) predicted by PLACE (Higo et al., 1999)

| Position | Copies | Response | Sequence | Cis-element | Gene  promotor | Site name |
| --- | --- | --- | --- | --- | --- | --- |
| 1195(-) | 1 | Sucrose | GAGAC | SURECOREATSULTR11 | *RafS1* | **Sugar** |
| 500(+) | 1 |  | TGACGT | TGACGTVMAMY |  |  |
| 1269(+),442(-) | 2 |  | TAACARA | AMYBOX1 |  |  |
| 1774(-),1564(+),  1346(-) | 4 |  | TGACT | WBOXHVISO1 |  |  |
| 1982(+) | 1 | Carbohydrate biosynthesis, ROS | MCACGTGGC |  |  |  |
| 608(+) | 1 | Sucrose | AATAGAAAA | SURE_1_STPAT_21_ | *RafS2* |  |
| 790(+) | 1 | Sucrose | AATACTAAT | SURE_2_STPAT_2_ |  |  |
| 1143(-) | 1 |  | TATCCAT | AMYBOX2 |  |  |
| 206 (+),328 (+),  478 (-),1244 (-),1278(-) | 5 |  | TGACT | WBOXHVISO1 |  |  |
| 1250(-) | 1 | Sucrose | TACTATT | SP8BFIBSP8BIB |  |  |
| 1923 (-), 2076(+),  2212 (-),354(+) | 4 | Sugars | TTATCC | SREATMSD |  |  |
| 2030 (+), 2394(-) | 2 | Sucrose | AATAGAAAA | SURE_1_STPAT_21_ | *RafS3* |  |
| 2226 (+), 592(+) | 2 | Sucrose | TACTATT | SP8BFIBSP8BIB |  |  |
| 1099(+),1611(-),  1638(-) | 3 |  | TGACT | WBOXHVISO1 |  |  |
| 1661(+),2457(-),  2515(-) | 3 | Sugars | TTATCC | SREATMSD |  |  |
| 1180(+) | 1 | Carbohydrate biosynthesis, ROS | MCACGTGGC |  |  |  |
|  |  |  |  |  |  |  |
| 916(-) | 1 |  | CCGAC | LTRECOREATCOR15 | *RafS1* | **Low temperature** |
| 1256(+) | 1 |  | CCGAAA | LTRE1HVBLT49 |  |  |
| 1780 (-) | 1 |  | ACCGAGA | DRE1COREZMRAB17 |  |  |
| 667 (+),2005(+) | 2 |  | CCGAC | LTRECOREATCOR15 | *RafS2* |  |
| 1848 (+),2029(+) | 2 |  | CCGAC | LTRECOREATCOR15 | *RafS3* |  |
| 1467(-) | 1 |  | CCGAAA | LTRE1HVBLT49 |  |  |
| 1280 (-) | 1 |  | ACCGAGA | DRE1COREZMRAB17 |  |  |
| 2028 (+) | 1 |  | ACCGAC | DRE2COREZMRAB17 |  |  |
| 2028 (+) | 1 |  | RCCGAC | DRECRTCOREAT |  |  |
|  |  |  |  |  |  |  |
| 1227(+) | 1 |  | CACCTG | RAV1BAT | *RafS1* | **ROS** |
| 1876(-) | 1 |  | AWTTCAAA | ERELEE4 |  |  |
| 135(+), 509(+), 74(-), 785 (+), 1014(+), 2153 (+), 2316(-),2383 (-) | 8 |  | AWTTCAAA | WBOXNTERF3 |  |  |
| 123(-), 497(-), 743(-), 1013(+), 224 (+), 2317 (-), 2384(-) | 7 |  | TTGAC | WBOXATNPR1 |  |  |
| 1,892(+) | 1 | Singlet oxygen | TTGACC | W box/EIRE |  |  |
| 611(+), 678(+), 2,252(+) | 3 | O_2_ Upregulated | AAGTCAAA | Defense response |  |  |
| 946(-) | 1 |  | CACCTG | RAV1BAT | *RafS2* |  |
| 141(+), 192(+), 221 (+), 308(-), 98 (+), 1106(+), 1256 (-), 2022(-),  2056 (-) | 9 |  | TGACY | WBOXNTERF3 |  |  |
| 191(+), 201(+),  309(-), 332(+),  1319(-), 1473(-),  2023(-), | 7 |  | TTGAC | WBOXATNPR1 |  |  |
| 498 (-) | 1 |  | AWTTCAAA | ERELEE4 |  |  |
| 862(+) | 1 | Peroxidase activity | AACGTG | CAT2-KD |  |  |
| 1708(+) |  |  | CGTGTG | ROSE2 | *RafS3* |  |
| 98(+), 285(+),  727(-), 1606(+), 1707(+),1742 (-), 1787(+),1954(-), 2026(+),2219(-), 2246(-) | 11 |  | TGACY | WBOXNTERF3 |  |  |
| 26(-), 211(+), 594(+), 728(-), 996(-), 1303(+)  1743(-), 2025 (+), 2113(-), 2202(+), 2247(-) | 11 |  | TTGAC | WBOXATNPR1 |  |  |
| 981 (+) | 1 |  | AWTTCAAA | ERELEE4 |  |  |
| 1070(+) |  | Singlet oxygen | ACGTGTC | ABRE |  |  |
|  | 1 | Singlet oxygen | TTGACC | W box/EIRE |  |  |
| 640(+) | 1 | ROS-specific | AGATATTT | SOD binding |  |  |
|  |  |  |  |  |  |  |

**Higo K, Ugawa Y, Iwamoto M, Korenaga T** (1999) Plant cis-acting regulatory DNA elements (PLACE) database: 1999. Nucleic acids research **27:** 297-300
