# Supplementary material for: An alternative pathway to plant cold tolerance in the absence of vacuolar invertase activity
Source: Plant J. 2022 Dec 22;113(2):327–41. doi: 10.1111/tpj.16049 (PMC10107833; doi:10.1111/tpj.16049)
Supplement: Supplementary file 7 — Table S6. List of primers used and their purpose. [file TPJ-113-327-s008.docx]

**Table S6.** List of primers used and their purpose

| **Primer (5'-3')** | **Name of primer** | **Gene name and/or purpose** |  |
| --- | --- | --- | --- |
| AGAGTCGACATAGCGATTGGACACATCATATAACGGCCGTTTTAGAGCTAGAAATAGCA | Inv-sgRNA9- *SalI* Fwd | Construction of 35S:Cas9‐AtU6:sgRNA‐nptII-sgRNA9 | 3 |
| GCTAAGCTTCGATCTAAAAAAAGCAC | U6-sgRNA HindIII Rev | Construction of 35S:Cas9‐AtU6:sgRNA‐nptII | 4 |
|  |  |  |  |
| ACCGGTGCGTGAGCTCGGATCCCTAGG | U6-sgRNA-AgeI Fwd | Construction of pSAT-Cas9-sgRNA(9) | 5 |
| ACCGGTCTGATCCAAGCTCAAGCTAAGCT | U6-sgRNA-AgeI Rev |  |  |
|  |  |  |  |
| ACCATCCTACCCGATGGTCA | VI_Fwd 2052 | *vinv*-mutant screening | 6 |
| CAGGTCAGCAGATTCACTAT | VI_Rev 2705 |  |  |
| AGCCTTCTTGGTAGCAGCTG | Cas9 -1_fw | CAS9 – to verify presence of the transgene | 7 |
| TTTCGTTGAGCAGCACAAGC | Cas9 -1_rv |  |  |
| GTAAAACGACGGCCAGT | sgRNA-9F | sgRNA – to verify presence of the transgene | 8 |
| GCTAAGCTTCGATCTAAAAAAAGCAC | sgRNA-9R |  |  |
|  |  |  |  |
| AAACTCCGCCTCCCATTAC | VI_Fwd 35 | *VInv* – qRT-PCR | 9 |
| AGGATCGGAAAGAAGGCTAC | VI_Rev 164 |  |  |
| AGTATGACGAATCTGGTCCTTCTAT | St_actin97_F | *Actin* –qRT-PCR | 10 |
| ACCCAACAATCAACTCTGCCCTCTC | St_actin97_R |  |  |
| ATTGGAAACGGATATGCTCCA | ef1α _Fwd | *Ef1α* – qRT-PCR | 11 |
| TCCTTACCTGAACGCCTGTCA | ef1α _Rev |  |  |
| AGATTGCCTGTTTGAAGACCCT | RafS1_F | PGSC0003DMG400018109 – qRT-PCR | 12 |
| ACCATCCTCCTCCTTGACAGTTA | RafS1_R |  |  |
| GATCCTTCTCGTGATGGTGTTAG | RafS2_F | PGSC0003DMG400030891 – qRT-PCR | 13 |
| TGTTATCGCCTCTGAGTTGGTT | RafS2_R |  |  |
| CTGGACAGCACGACTTTAATCTT | RafS3_Fw | PGSC0003DMG400022258 – qRT-PCR | 14 |
| CACCTTGACAGTTGAATACACCG | RafS3_Rv |  |  |
| GGTGTTATTGCGAATCGAGAAGGA | MIPS1_Fw | PGSC0003DMG400021831 –qRT-PCR | 15 |
| CCAAATACTACGTCGTCTGGGTT | MIPS1_Rv |  |  |
| GGCTGAAATATCTCCTTCCACCT | MIPS2_Fw | PGSC0003DMG400027916 – qRT-PCR | 16 |
| CAACCAGCACTGACTTCATCTTG | MIPS2_Rv |  |  |
| CCGTGGAGGTACACAGGGAA | GolS1_Fw | PGSC0003DMG400005216 – qRT-PCR | 17 |
| GTCCAGAGACTCGTCGTTGT | GolS1_Fw |  |  |
| TTTATATGCGGTGGCGGACT | GolS2_Fw | PGSC0003DMG400046135 –qRT-PCR | 18 |
| GGTGGGTGGGGTAACTTTGA | GolS2_Rv |  |  |
| GAACCTGTTTATCCTCCTGAGA | GolS3_Fw | PGSC0003DMG400003522 –qRT-PCR | 19 |
| TCCACAAACTCCCAAATACGG | GolS3_Rv |  |  |
| TCTATTGGAAGAGTTGGGGATGAT | StS1_Fw | PGSC0003DMG400009017 –qRT-PCR | 20 |
| GTAGCCAGTACACTCCATTCGG | StS1_Rv |  |  |
| CAAGGTGTGTGGGAAGGTGT | StS2_Fw | PGSC0003DMG400000513 –qRT-PCR | 21 |
| GGTGATTGGGTCATCGTCGT | StS2_Rv |  |  |
