# Supplementary material for: An alternative pathway to plant cold tolerance in the absence of vacuolar invertase activity
Source: Plant J. 2022 Dec 22;113(2):327–41. doi: 10.1111/tpj.16049 (PMC10107833; doi:10.1111/tpj.16049)
Supplement: Supplementary file 8 — Table S7. Solutions used for protoplast extraction. [file TPJ-113-327-s006.docx]

**Table S7.** Solutions used for protoplast extraction

**Enzyme solution**

| Final volume  50 mL | Cat. number | Manufacturer | Mw  g/mol | Compound |
| --- | --- | --- | --- | --- |
| 12.5 mg | C1301 | Duchefa |  | Casein hydrolysate |
| 8.5 mg | S0522 | Duchefa | 156 | KH_2_PO_4_ |
| 15 mg | P0515 | Duchefa | 74.5513 | KCI |
| 30 mg | C3306 | Sigma | 147 | CaCl_2_ x 2H_2_O |
| 30 mg | A0501 | Duchefa | 80.043 | NH_4_NO_3_ |
| 95 mg | P8291 | Sigma | 101.11 | KNO_3_ |
| 15 mg | 63138 | Sigma | 246.5 | MgSO_4_ · 7H_2_O |
| 6 g | S0809 | Duchefa | 342.3 | Sucrose |
| 500 μL |  |  |  | 100X Fe/EDTA |
| 500 μL |  |  |  | 100X Organic acids |
| 100 μL |  |  |  | B5 vitamins (X500) |
| 500 μL |  |  |  | 100X Gamborg's microelemants |
| 150 mg | M8002 | Duchefa | - | Macerozyme R10 |
| 500 mg | C8001 | Duchefa | - | Cellulase R10 |

**100x Fe/EDTA**

| Final volume  100 mL | Cat. number | Manufacturer | Mw  g/mol | Compound |
| --- | --- | --- | --- | --- |
| 140 mg | E5134 | Sigma | 372.2 | Na_2_EDTA |
| 190 mg | 1.3965 | Merck | 278 | FeSO_4_ · 7H_2_O |

**100X Organic acids**

| Final volume  100 ml | Cat. number | Manufacturer | Mw  g/mol | Compound |
| --- | --- | --- | --- | --- |
| 100 mg | P5280 | Sigma | 110 | Pyruvic acid |
| 200 mg | F8509 | Sigma | 116.08 | Fumaric acid |
| 200 mg | C1303 | Duchefa | 210.1 | Citric acid monohydrate |
| 200 mg | M1315 | Duchefa | 134.1 | DL-malic acid |

**B5 vitamin mix– 500X**

| Final volume  100 mL | Cat. number | Manufacturer | Mw  g/mol | Compound |
| --- | --- | --- | --- | --- |
| 50 mg | N0611 | Duchefa | 123.1 | Nicotinic acid |
| 50 mg | P9755 | Sigma | 205.6 | Pyridoxine hydrochloride |
| 50 mg | T1270 | Sigma | 337.2 | Thiamine hydrochloride |
| 5 g | I0609 | Duchefa | 180.2 | Myo-inositol |

**Gamborg's (B5) microelements stock 100X**

| Final volume  1 L | Cat. number | Manufacturer | Mw  g/mol | Compound |
| --- | --- | --- | --- | --- |
| 2.5 mg | 2539 | Merck | 237.9 | CoCl_2_ · 6H_2_O |
| 2.184 mg | C3036 | Sigma | 249.6 | CuSO_4_ · 5H_2_O |
| 300 mg | B0503 | Duchefa | 61.8 | H_3_BO_3_ |
| 75 mg | P0518 | Duchefa | 166 | KI |
| 1 g | M7634 | Sigma | 169 | MnSO_4_ · H_2_O |
| 25 g | S0522 | Duchefa | 156.0 | NaH_2_PO_4_ · 2H_2_O |
| 25 mg | S0525 | Duchefa | 241.9 | NaMoO_4_ · 2H_2_O |
| 200 mg | 8883 | Merck | 287.5 | ZnSO_4_ · 7H_2_O |

**Wash (W5)**

| Final volume  400 mL | Cat. number | Company | Mw  g/mol | Compound |
| --- | --- | --- | --- | --- |
| 2.99 g | S3014 | Sigma | 58.4 | NaCl |
| 6.15 g | C3306 | Sigma | 147.01 | CaCl_2_ · 2H_2_O |
| 2.5 mL (from 0.2 M stock) | M1503 | Duchefa | 213.2 | MES |
| 2.5 mL (from 2 M stock) | P5405 | Sigma | 74.55 | KCl |
